# The landscape of facial processing applications in the context of the European AI Act and the development of trustworthy systems

**DOI:** 10.1038/s41598-022-14981-6

**Published:** 2022-06-23

**Authors:** Isabelle Hupont, Songül Tolan, Hatice Gunes, Emilia Gómez

**Affiliations:** 1 Joint Research Centre, European Commission, Seville, Spain; 2 Department of Computer Science and Technology, University of Cambridge, Cambridge, UK

**Keywords:** Computational science, Computer science, Software

## Abstract

This work focuses on *facial processing*, which refers to artificial intelligence (AI) systems that take facial images or videos as input data and perform some AI-driven processing to obtain higher-level information (e.g. a person’s identity, emotions, demographic attributes) or newly generated imagery (e.g. with modified facial attributes). Facial processing tasks, such as face detection, face identification, facial expression recognition or facial attribute manipulation, are generally studied as separate research fields and without considering a particular scenario, context of use or intended purpose. This paper studies the field of facial processing in a holistic manner. It establishes the landscape of key computational tasks, applications and industrial players in the field in order to identify the 60 most relevant applications adopted for real-world uses. These applications are analysed in the context of the new proposal of the European Commission for harmonised rules on AI (the AI Act) and the 7 requirements for *Trustworthy AI* defined by the European High Level Expert Group on AI. More particularly, we assess the risk level conveyed by each application according to the AI Act and reflect on current research, technical and societal challenges towards trustworthy facial processing systems.

## Introduction

Automatic facial processing refers to systems that take facial images or videos as input data and perform some algorithmic processing to obtain higher-level information (e.g. a person’s identity, emotions, demographic attributes, etc.) or newly generated imagery (e.g. with modified facial attributes). It is one of the most studied areas in various Artificial Intelligence (AI) research communities including computer vision, biometrics and affective computing. While both academia and industry have been increasingly working on technically improving and creating facial processing systems, legal and ethically questionable aspects have just started to be discussed.

There is a public debate around the need for *Trustworthy AI*, an approach to AI that considers legal and ethical aspects right from the start when AI systems are developed. Part of the European Commission’s (EC) response to this debate constitutes the creation of a High Level Expert Group for ethical guidelines on AI^1^ (AI HLEG). The guidelines support a responsible human-centric approach to the development of AI and put into place *7 requirements for Trustworthy AI*, which have to be continuously evaluated throughout an AI system’s life cycle and for which implementation and relevance strongly depend on the specific application, namely: (1) human agency and oversight; (2) technical robustness and safety; (3) privacy and data governance; (4) transparency; (5) diversity, non-discrimination and fairness; (6) societal and environmental well-being; and (7) accountability.

The EC’s efforts towards Trustworthy AI culminated on April 21 2021 with the publication of the AI Act, a proposal for a regulation laying down harmonised rules on AI^2^. Rather than the AI techniques specifically (i.e. algorithms, models, architectures), the new European AI Act focuses on particular AI applications and the risk of their intended use. The AI Act establishes 4 risk levels, from highest to lowest: (1) “Unacceptable” risk or prohibited use cases, which cover harmful uses of AI or uses that contradict ethical values; (2) “High-risk” use cases, which are identified through a list of “high-risk” AI application areas that may create an adverse impact on people’s safety, health or fundamental rights; (3) “Transparency” risk use cases, subject to a limited set of transparency rules, e.g., informing people that they are exposed to such a system; and (4) “Minimal” or no risk use cases, which cover all other AI systems that can be developed and used in the EU without additional legal obligations than the already existing legislation. Each risk level has a clear definition and a strict set of requirements to be fulfilled, which are aligned with the 7 requirements of Trustworthy AI.

**Table 1:** Definitions as provided in the European Commission’s AI Act proposal that are particularly relevant for categorising facial processing systems and applications. Note that the AI Act is currently under discussion with European co-legislators (as of June 2022) and these definitions might be subject to change.

Concept	Definition as in the AI Act	Article
Biometric data	Personal data resulting from specific technical processing relating to the physical, physiological or behavioural characteristics of a natural person, which allow or confirm the unique identification of that natural person, such as facial images or dactyloscopic data.	Article 3(33)
Emotion recognition system	AI system for the purpose of identifying or inferring emotions or intentions of natural persons on the basis of their biometric data.	Article 3(34)
Biometric categorisation system	AI system for the purpose of assigning natural persons to specific categories, such as sex, age, hair colour, eye colour, tattoos, ethnic origin or sexual or political orientation, on the basis of their biometric data.	Article 3(35)
Remote biometric identification system	AI system for the purpose of identifying natural persons at a distance through the comparison of a person’s biometric data with the biometric data contained in a reference database, and without prior knowledge of the user of the AI system whether the person will be present and can be identified.	Article 3(36)
“Real-time” remote biometric identification system	Remote biometric identification system whereby the capturing of biometric data, the comparison and the identification all occur without a significant delay. This comprises not only instant identification, but also limited short delays in order to avoid circumvention.	Article 3(37)
“Post” remote biometric identification system	Remote biometric identification system other than a “real-time” remote biometric identification system.	Article 3(38)

The AI Act has many explicit and implicit references to facial processing. From the set of 44 definitions in its Article 3, the 6 presented in Table 1 are particularly relevant for categorising facial processing tasks and applications. They are mostly related to biometrics (identification and categorisation of persons) and emotion recognition, and are considered at different risk levels including some “prohibited” and “high-risk” uses. For instance, according to Article 5(1d), *the use of “real-time remote” biometric identification systems in publicly accessible spaces for the purposes of law enforcement* is considered a prohibited practice with some exceptions such as the targeted search for potential victims of crime (including missing children), the prevention of a terrorist attack or the prosecution of a suspect of a criminal offence. According to Annex III(1), all AI systems intended to be used for the “real-time” and “post” remote biometric identification of natural persons are considered “high-risk”.

This annex also categorises as “high-risk” the AI systems intended to recognize a person’s emotions as a polygraph in the context of law enforcement (Annex III(6b)) and migration, asylum and border control management (Annex III(7a)). In other scenarios, a “transparency” risk applies to emotion recognition and biometric categorisation systems according to Article 52, such as facial processing applications aimed at assigning individuals to specific categories (e.g. based on age, gender, ethnicity) or to manipulate video content (as in *deepfakes*). However, Article 52 does not apply to AI systems used for biometric categorisation which are permitted to detect, prevent and investigate criminal offences. There might be situations where emotion recognition or biometric categorisation systems are also exploited in some other “high-risk” use cases included in Annex III, e.g. in recruitment (Annex III(4a)) or for access to educational institutions (Annex III(3a)), but they are not mentioned specifically in the legal text. In addition, a facial processing system could be considered as a safety component of a product (e.g. a system integrated in a car that detects a driver’s drowsiness and undertakes a safety action) and be deemed “high-risk” according to Article 6(1a).

**Figure 1:**
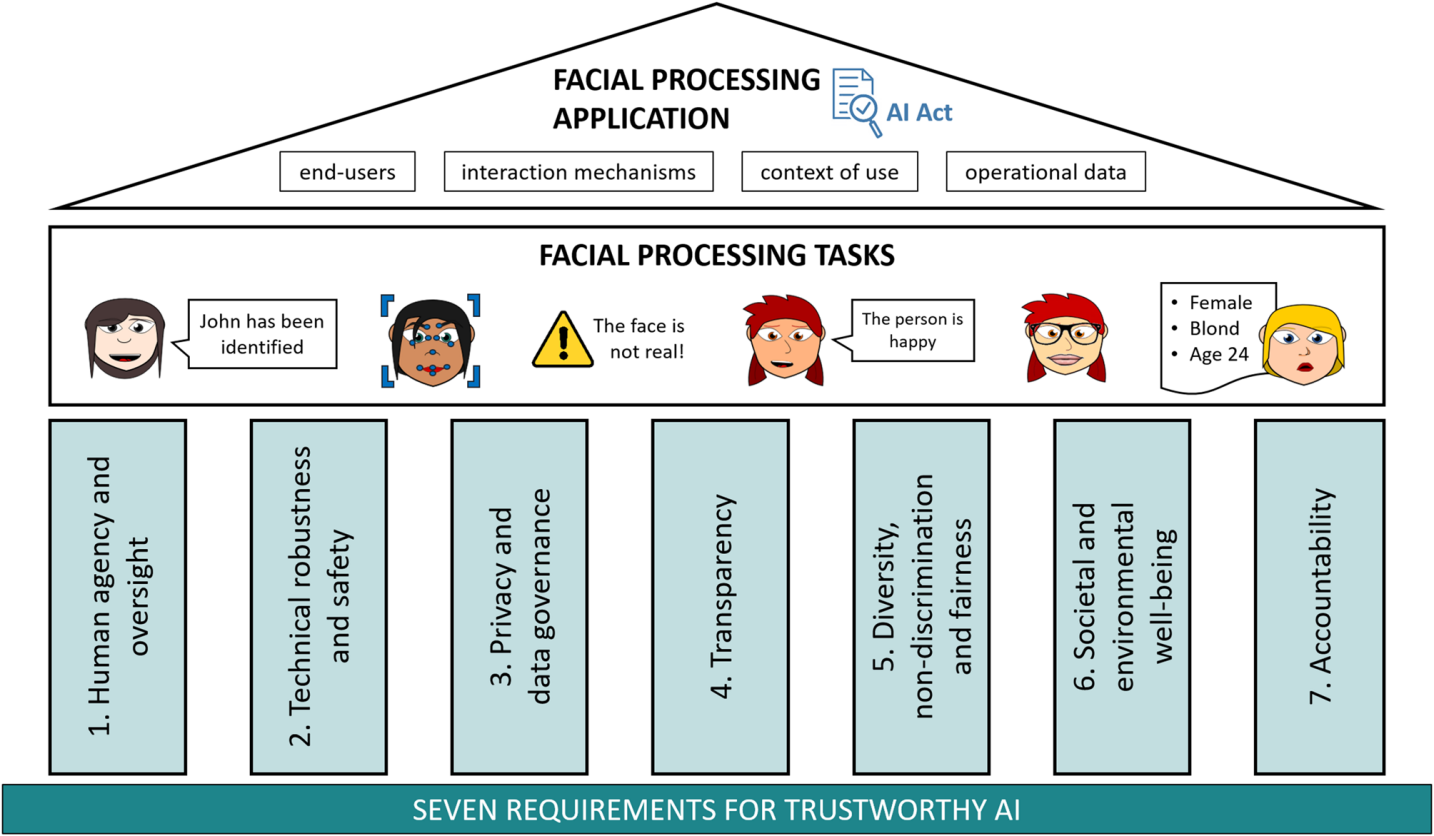
Objective of this work: establishing the landscape of facial processing tasks and applications in the context of the 7 requirements for Trustworthy AI and the new European AI Act. The requirements for Trustworthy AI are the 7 pillars upon which facial processing tasks must been built. A facial processing application is a real-world use case utilising one or more facial processing tasks, with a particular intended purpose and in a concrete context of use, being the object of the AI Act. Face drawings are courtesy of *Pixabay* (https://pixabay.com).

This paper assesses for the first time the field of facial processing in the context of the 7 requirements for Trustworthy AI and the European AI Act, as illustrated in Fig. 1. For this purpose, it first establishes the landscape of facial processing computational tasks having caught research efforts in the last 10 years. Then, the 60 more relevant real-world applications making use of such computational tasks in a particular context and with a concrete intended use are identified, together with related key industrial players, and the risk level of each application is assessed according to the AI Act. Finally, it reflects on current research, technical and societal challenges that need to be addressed to increase trust in these facial processing applications.

## Background: facial processing computational tasks and their technology transfer to real-world applications

In this work, we use *facial processing* as an umbrella term for different automatic facial processing tasks that are generally considered as independent topics. In the following, we provide an overview of such tasks and explain how they can be brought to real-world applications.

### Most relevant facial processing tasks

To identify the most relevant facial processing tasks, we compiled the face-related topics appearing in the call for papers of the IEEE International Conference on Automatic Face and Gesture Recognition^3^ and the International Joint Conference on Biometrics^4^ since 2010. These are the most prominent forums for presenting the latest research in facial processing and biometrics, respectively, with a strong presence of academia but also of research centres and industry.

**Figure 2:**
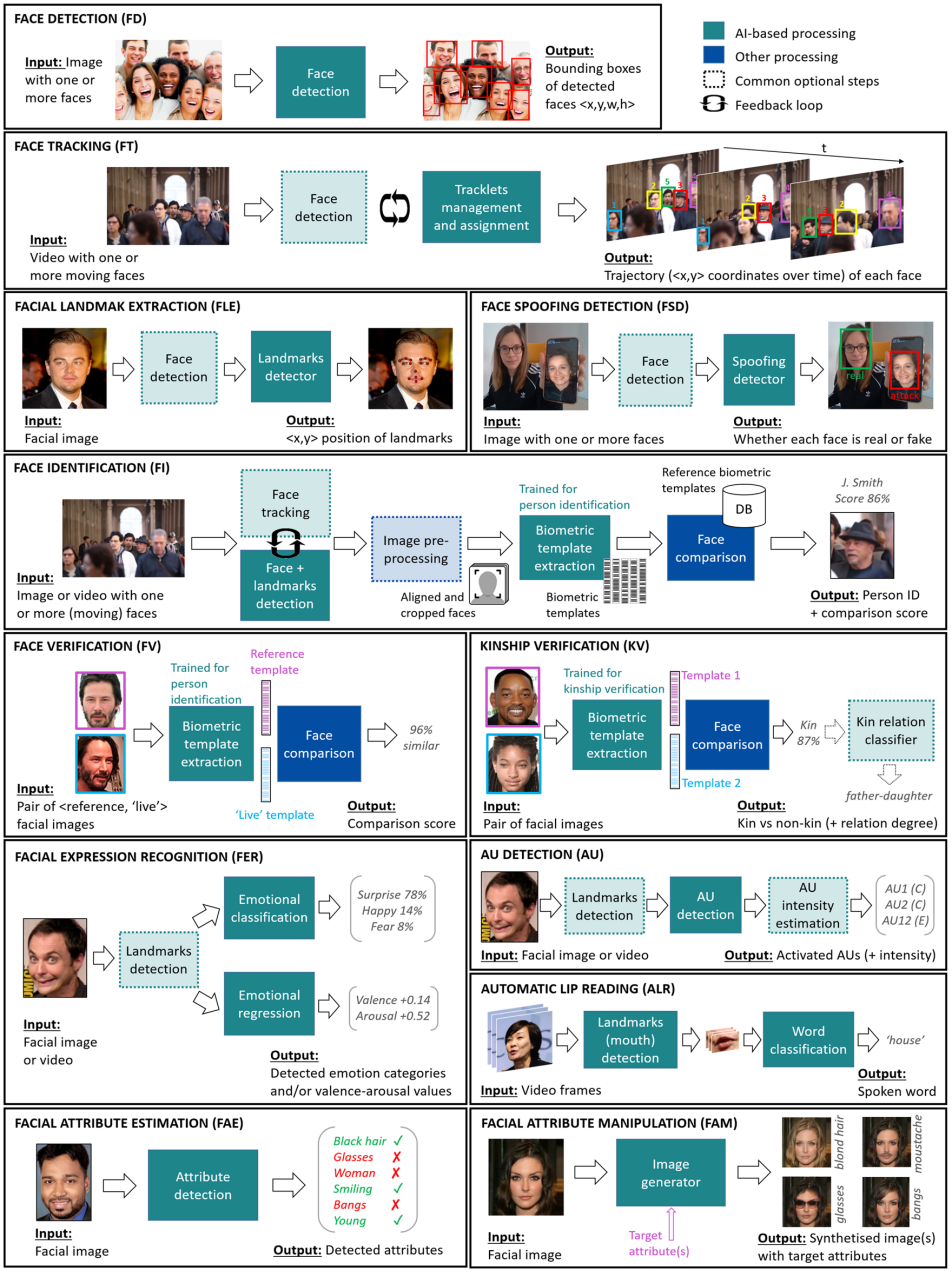
Inputs, outputs and most commonly used computational pipelines for the facial processing tasks studied in this work, identified by their corresponding acronym.

We found the following 12 facial processing tasks having attracted researchers’ interest in the last decade: (1) face detection; (2) face tracking; (3) facial landmark extraction; (4) face spoofing detection; (5) face identification; (6) face verification; (7) kinship verification; (8) facial expression recognition; (9) Action Unit detection; (10) automatic lip reading; (11) facial attribute estimation; and (12) facial attribute manipulation. Table 2 describes each task in detail and Fig. 2 further illustrates related computational pipelines.

It can be seen that different facial processing tasks can build on each other. For instance, face detection can be performed in isolation but it is also a common part in more complex tasks such as face tracking or face identification. Also, feedback loops (iteratively using one task’s output as input to another, and vice-versa) are a common practice. For example, face detection and facial tracking tasks could be used together to improve one another (known as “tracking-by-detection”^5^).

**Table 2:** Description of most relevant facial processing tasks in AI research. Each task is assigned an acronym which is used as reference throughout the paper.

Task	Acronym	Description
Face detection	FD	Determines the presence of faces in an image and, if present, returns the location and extent of each face^6^.
Facial landmark extraction	FLE	Locates facial salient features, such as points around the eyes, nose and mouth^7^.
Face tracking	FT	Tracks (i.e. follows) the position of each face appearing in a video, from the point it enters until the point it leaves the scene^5^. Each tracked face is assigned a random identifier (generally in numeric format) during the process.
Face identification	FI	Carries out a one-to-many (1:N) query for a “live” detected face against a database of known faces (e.g. a blacklist of N persons). It involves the extraction of a biometric template for each detected face, i.e. a small-size feature vector containing the most relevant facial information^8^, and a “face comparison” (or “face matching”) process comparing each biometric template to those from images pre-enrolled in the database for identification purposes. FI can be done remotely, as it does not require active cooperation of the persons^9^.
Face verification	FV	Also called face authentication, it performs a one-to-one (1:1) query for a “live” detected face against a reference facial image of a known person^10^. It verifies that this person is who she claims to be, and thus needs some cooperation from her (e.g. the person must be willing to pose in front of a camera for unlocking a smartphone or at airport check-ins).
Kinship verification	KV	Aims at finding out whether there is a kin (i.e. family) relationship or not between given persons by analysing their facial images^11^. It might additionally detect the relation degree, e.g. brother-sister, father-child, grandmother-grandson and so on.
Face spoofing detection	FSD	It is also known as *presentation attack detection* or *anti-spoofing*. Face spoofing is a false acceptance in which attackers present a fake evidence to the biometric system (in this case, a face identification/verification system). Face spoofing attacks can be of different nature, such as photo attacks, video attacks or 3D mask attacks^12^. FSD consists on detecting whether the input face presented to the system is real (i.e. a real “live” human face is in front of the camera) or fake (e.g. an image or video attack is presented instead).
Facial expression recognition	FER	“Facial expression recognition” and “facial emotion recognition” have been used interchangeably in the literature. The FER task aims at automatically detecting expressions of emotion from a person’s facial image or video^13^.
Action Unit detection	AU	Action Units (AUs) encode movements of facial muscles and their intensity according to the Facial Action Coding System (FACS)^14^. Examples of AUs include: “brow lowerer” (AU4), “cheek raiser” (AU6) or “lip corner puller” (AU12). AU detection provides an objective and fine-grained description of a person’s facial behaviour.
Facial attribute estimation	FAE	Recognises whether certain attributes are present in given facial images^15^. Examples of such facial attributes include: hair/eye colour, mustache, beard, wavy hair, bangs, pointy nose, big lips, oval face, eyeglasses, wearing hat, etc. The estimation of demographic attributes such as age, gender and ethnicity are also considered part of the FAE task.
Facial attribute manipulation	FAM	Synthesising or removing desired attributes from the original facial image^15^. For example, it might be used to put glasses on a given face, remove beard, ageing the person or manipulating AUs or facial expressions (e.g. transforming a smile into a neutral expression) in the most photo-realistic manner. FAM is at the origin of now called “deepfakes”, which are hyper-realistic videos using face swaps that leave little evidence of manipulation^16^.
Automatic lip reading	ALR	Decode speech (spoken words) exclusively by analysing facial (lip/mouth region) images, i.e. mimicking the human capability to perform lip reading^17^.

### From facial processing tasks to applications

Building facial processing applications ready to be deployed in real-world scenarios requires more than the mere combination of computational tasks: the *technology transfer* gap has to be bridged. Technology transfer is the process of translating results from scientific and technological research from the lab to real production. It requires various implementation efforts and design choices at different levels. Besides pure algorithmic developments, other factors must be carefully assessed, including: the context of use (e.g. law enforcement, entertainment), the integration with specific software and hardware (operating system, GPU, CPU), data gathering procedures (connection to camera sensors and databases), the target users of the system (e.g. experts, the wide public), the population on which the system will be applied (e.g. pedestrians, factory workers), real-time constraints, user interfaces, and interaction mechanisms needed (e.g. human oversight strategies to access and refine the system’s output).

For example, a face identification system might be used for many different applications, e.g. for video-surveillance or face-to-face interaction with a robot. These applications might have the same AI core, but they implement different components on top to allow for specific interactions (e.g. through a PC screen or a robotic embodiment), visualizations and human oversight mechanisms.

The EU’s AI Act requirements apply to the final use of a system in the context of its “intended purpose”, which is defined as *the use for which an AI system is intended by the provider, including the specific context and conditions of use*. Thus, to explain facial processing in the context of the AI Act, we conducted a comprehensive assessment of existing applications.

## Methodology for the selection of facial processing applications

The facial processing tasks reviewed above have been applied worldwide in different scenarios, both by academic researchers and industry players. To provide a comprehensive collection of real and relevant use cases we have adopted the following methodology.

### Application selection from scientific papers

To find the scientific publications related to facial processing in the last 10 years (January 2012 to December 2021), we conducted a keyword search for the 12 tasks in Table 2 on the Web of Science platform^18^. From the 37,194 obtained papers we identified only 211 making specific mention to a final application and presenting some concrete experiments, proof of concepts or deployments in that direction. This demonstrates that the majority of facial processing research papers rarely addresses real use cases.

### Application selection from companies’ portfolios

We identified key AI companies worldwide performing research and development on facial processing and/or having related products in their portfolio. To do so, we combined different sources of information. First, we searched for company authors (i.e. company affiliations) in our collection of scientific papers. Second, we consulted the reports by the US National Institute for Standards and Technology (NIST^19^), which is a reference source for monitoring key vendors of facial processing technology. Third, we consulted the Affective Computing Commercial Products Database^20^ by the Association for the Advancement of Affective Computing (AAAC) for companies with products related to the recognition of facial expressions of emotions. Finally, we performed a comprehensive search on the web and the professional social network LinkedIn. The resulting list of 183 identified companies is presented in Supplementary Table 6. For each identified company, we looked for specific applications (use cases, success stories, real deployments, case studies and integrations) in their websites.

### Final selection and annotation of applications

We considered only those use cases that have at least three related scientific papers or one company with related products in the market. For each identified source, we manually labelled the facial processing computational tasks involved and assigned a set of application areas from the list in Table 3. The eight top rows in Table 3 correspond to the “high-risk” areas covered in Annex III of the AI Act and the subsequent rows are other areas of interest we identified during our use case assessment. We finally associated a set of reference academic papers and key companies (distinguishing between SMEs and large firms) to each use case, and assessed its risk level according to the AI Act. It is important to note that the risk assessment was performed by the authors based on their interpretation of the AI Act as of June 2022. As the AI Act is still under discussion with European co-legislators, this risk assessment might be subject to change in the future.

**Table 3:** Areas considered for the assessment of facial processing applications. Eight top rows correspond to the “high-risk” areas mentioned in the AI Act, under Annex III. The number of use cases related to each area are also shown, per type of system (*BI* Biometric identification, *BC* Biometric Categorisation, *ER* Emotion Recognition, and *OT* Other) and in total. The most frequent application area is in bold and second most frequent underlined.

Code	Area	Number of use cases				
		BI	BC	ER	OT	Total
BIC	Biometric identification and categorisation of natural persons	**20**	**7**	0	0	27
MCI	Management and operation of critical infrastructure	8	0	0	1	9
EDU	Education and vocational training	3	1	3	0	7
EMP	Employment, workers management and access to self-employment	5	1	1	0	7
SER	Access to and enjoyment of essential private services and public services benefits	2	1	0	0	3
LE	Law enforcement	12	2	2	8	24
MIG	Migration, asylum and border control management	2	1	1	0	4
JUS	Administration of justice and democratic processes	1	1	1	3	6
ENT	Entertainment and leisure	7	2	**10**	**10**	**29**
MKT	Marketing and retail	4	4	5	2	15
CUL	Culture, art and heritage	0	0	3	1	4
CLI	Clinical use in medicine and healthcare	6	2	4	8	20
FIN	Finances and banking	4	1	0	0	5
SOC	Social assistance	1	1	2	2	6
VSU	Video-surveillance for security	9	2	1	6	18
TRA	Transportation and mobility	4	1	2	0	7
TOU	Tourism, hotels and restaurants	3	2	1	1	7
IND	Industry and logistics	4	0	0	0	4
POL	Politics	2	0	1	1	4

## Identified facial processing applications

This section presents the resulting list of 60 facial processing applications, as shown in Table 4. They are divided into four categories, depending on the type of system they implement according to AI Act’s definitions: (1) Biometric Identification (BI), with 20 use cases; (2) Biometric Categorisation (BC), with 7 uses cases; (3) Emotion Recognition (ER), with 18 use cases; and Other (OT) applications, with 15 use cases. Further information on use cases, including academic references and company names, can be found in Supplementary Tables 2–5.

**Table 4:**
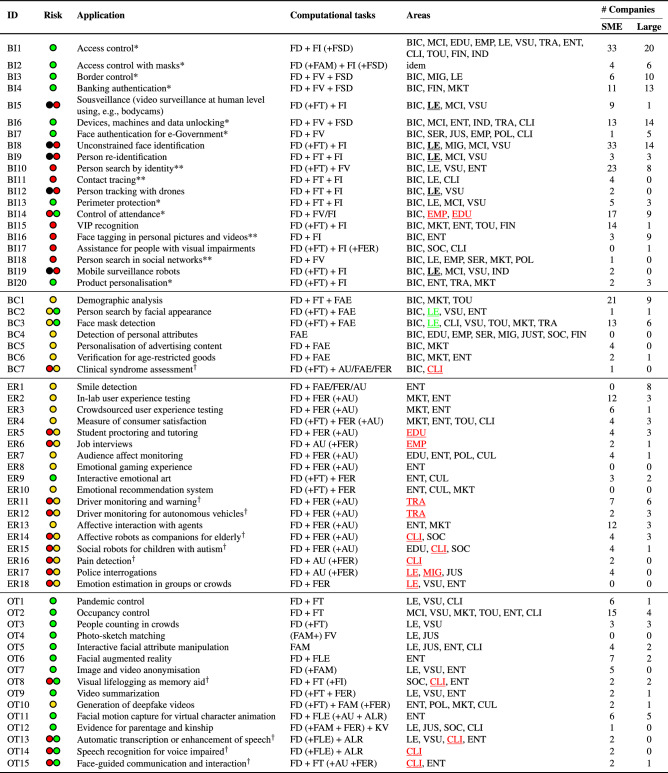
Details on the 60 identified use cases. Application identifier (ID) starts with BI for *Biometric Identification*; BC for *Biometric Categorisation*; ER for *Emotion Recognition*; and OT for *Others*. BI applications marked with * and ** indicate “non-remote” and “post” BI, respectively. Applications marked with ^†^ could possibly be “high-risk” when the AI system is a safety component or part of a medical device or machine. The risk levels conveyed by each use case according to the AI Act are marked with coloured circles as follows:  “unacceptable risk” or prohibited practice;  “high-risk”;  “transparency” risk; “minimal” risk. Some use cases may exceptionally entail two different risk levels, depending on their application area, because of exceptions stated in the legal text. In these cases, we have underlined the exception area in its corresponding colour. Please note that risk labels have been assigned by the authors based on their own interpretation of the AI Act. At the time of writing this paper (June 2022), the AI Act is under discussion with the European co-legislators and the assignment of risk levels might be subject to change in the future.

### Most established applications

We find that the most established Biometric Identification (BI) applications are unconstrained face identification, access control, surveillance and person search, which are mostly deployed in law enforcement and security (video-surveillance and management of critical infrastructure). Interest in both on-site and virtual control of school/workplace attendance is also very high, probably favoured by the Covid-19 pandemic. Surprisingly, the recognition of VIP customers (for instance, when entering a shop or hotel to greet them by their name, offer discounts, etc.) is also one of the BI applications most present in the market. By contrast, we only find one company addressing a more societal well-being oriented application such as the assistance for people with visual impairments.

Biometric Categorisation (BC) applications are dominated by demographic analysis for marketing purposes, e.g., to obtain aggregated statistics on customers’ shopping habits by age, gender and ethnicity. The detection of facial masks also has a high market presence due to the need for Covid-19 prevention measures. Decision-making on the basis of detected personal attributes such as sexual or political orientation, gender and ethnicity, has remained in few controversial academic works. The automatic facial assessment of syndromes such as autism or psychosomatic disorders is starting to emerge as a new application in academia and start-ups.

Facial processing for Emotion Recognition (ER) is mostly exploited in entertainment and marketing domains. Applications for user experience testing, both in the lab and via crowdsourcing, and for consumer experience and satisfaction assessment have a strong market presence. Facial expression recognition for the affective interaction with social virtual agents and robots is also very popular, mostly in these two domains, but also with some societal and clinical applications such as accompanying the elderly or helping children with autism to better manage their emotions. Interestingly, ER is the category with more sector-specific applications. Examples include transport (driver monitoring), medicine (pain detection), criminal justice (police interrogations), education (online student proctoring) and employment (job interviews).

Regarding other (OT) applications, pandemic needs have opened the door to use cases such as occupancy control, people counting and pandemic control (e.g. social distance monitoring). Virtual and augmented reality are also popular for the entertainment of the general public. Applications with a strong societal and clinical impact exist but have yet a timid market presence, such as the use of face detection for visual lifelogging as memory aid, speech recognition for voice impaired or face-guided interaction for people with mobility problems (e.g. to control a wheelchair).

Overall, it is important to highlight the role of SMEs in the landscape of facial processing applications (c.f. two last columns in Table 4). SMEs account for 66% of the total number of companies and are the ones contributing the most towards positive social impact applications as mentioned above. For more details see Supplementary Fig. 4.

### Risk levels according to the AI Act

Table 4 illustrates the risk level of each identified application according to the authors own interpretation of the AI Act. In the AI Act, higher risk levels come with more requirements and obligations for system providers.

We find that the Biometric Identification (BI) category is the only one comprising some “high-risk” use cases that could even be “prohibited” under certain application areas. This occurs for “remote” and “real-time” BI when used for law enforcement purposes (BI5, BI8, BI9, BI12 and BI19), which is in principle prohibited with exceptions including the targeted search for missing persons or the prevention of terrorist attacks (c.f. AI Act’s Article 5(1d)). “Remote” and “real-time” BI, as in BI15 and BI 17, is “high-risk” but not “prohibited” as not intended to be used for law enforcement. “Remote” BI when performed in a “post” manner is “high-risk” but not “prohibited” either, even for LE purposes (BI10, BI11, BI16, BI18). On the other side, “non-remote” BI use cases do not fall under AI Act’s Annex III(1a) and are considered “minimal” risk (BI1, BI2, BI4, BI6, BI7, BI13, BI20), with the exception of *control of attendance* use case BI14 that could eventually be “high-risk” under Annex III(3b/4b) if used in education/employment with the purpose of penalising students/workers (e.g. if not present in a course or at the workplace).

The Biometric Categorisation (BC) and Emotion Recognition (ER) categories comprise mostly “transparency” risk applications. There are a few exceptions for the BC category. On the one hand,“minimal” risk applies to BC2 and BC3 when used by law enforcement bodies to investigate criminal offences (Article 52(3)). On the other hand, the *clinical syndrome assessment* use case BC7 could eventually be part of the safety component of a medical device and as such be “high-risk” according to Article 6(1a). The latter Article also applies to several ER use cases, namely those that could be a safety component of a car (ER11 and ER12) or part of a medical device (ER14, ER15, ER16). The ER category has other “high-risk” exceptions that might occur when using the recognition of facial expressions of emotions for education (ER5), recruitment (ER6), law enforcement (ER17, ER18) and migration (ER17) as in AI Act’s Annex III(3b/4a/6b/7a). The only “minimal” risk application under category ER is the *interactive emotional art* use case ER9, that is free from “transparency” obligations in light of the *right to freedom of the arts* (Article 52(3)).

As for the Other (OT) category, it is mostly linked to “minimal” risk use cases. *Deepfakes* (OT10) is the only OT application involving a “transparency risk” as explicitly mentioned in Article 52(3) of the legal text. Additionally, four OT use cases could eventually be “high-risk” when used as a safety component of medical devices (OT8, OT13, OT14 and OT15).

It is noteworthy that some applications with “high-risk” level, such as *unconstrained face identification* (BI8), *person search by identity* (BI10) and *control of attendance* (BI14) have already a strong presence in the market and real operational settings. Nevertheless, these applications and their related computational tasks still need considerable research efforts towards compliance with the 7 requirements for Trustworthy AI and the AI Act, as will be discussed in the next section.

## Challenges towards trustworthy facial processing applications

In this section, we discuss the main challenges that need to be addressed when working towards trustworthy facial processing applications. For this purpose, we elaborate on existing research efforts and discuss how they relate to the 7 requirements for Trustworthy AI.

### Facial datasets: the problem of data quality, bias and governance

Datasets are the key element to train, test and validate facial processing systems. Efforts towards improving data quality, data fairness (i.e. data that does not create or reinforce bias) and data handling contribute to multiple Trustworthy AI requirements. In the following, we summarize and discuss the main use and characteristics of the current state-of-the-art facial datasets, which are further detailed in Supplementary Table 1.

First, high quality datasets and ideally “error free” data annotations contribute to the requirement (2) “technical robustness”. Deep learning models for facial processing are usually trained on large datasets with up to millions of facial images. Developers obtain these large datasets through web scraping for faces of celebrities such as actors, politicians, athletes or singers. Datasets for face identification and verification are the largest and more unconstrained in terms of head pose, facial occlusions, illumination and background, theoretically reproducing real-life (“in the wild”) conditions. However, they are also the noisiest, i.e., many images have incorrect labels. For example, the original version of the widely used MS-Celeb-1M^21^ has more than 50% mislabelled samples as a result of massive web scraping^22^. Studies have shown a clear degradation in face identification performance when the noise level increases. For instance^23^, demonstrates that a manual correction of 10% of mislabeled samples produces roughly similar results to doubling the dataset size. Great efforts have been devoted to the development of clean –but much smaller and sometimes taken in controlled lab settings– facial datasets, such as IMDB-Face^22^, CelebA^24^ and BP4D^25^. However, cleaning is a tedious and time-consuming task requiring full or partial manual checks. Recently, the authors of the WebFaces42M dataset^26^ have presented a fully automated cleaning methodology achieving a noise level below 10% in their database of 42 million images. Nevertheless, 10% noise (i.e. 4.2 million mislabelled images ) is still very far from the AI Act’s requirement of “error free” datasets (Article 10(3)).

Another Trustworthy AI requirement that should be addressed at the data stage of an algorithm is (5) “diversity, non-discrimination and fairness”. Facial datasets are extremely imbalanced with respect to demographic factors. One reason for this imbalance lies in the data generating process, i.e. web-scraping celebrity faces, which causes strong biases towards western, white, “young and beauty” faces with heavy make up. Most comprehensive datasets contain a vast majority of white male faces, e.g., WebFaces42M has 70% white and 38% female faces. More importantly, annotations of age, gender and -especially- ethnicity and skin colour are neither available nor provided in most cases, suggesting that these facial images have been collected at mass, ignoring demographic distributions. Also, the few datasets providing facial attribute annotations do not match general appearance distributions of real-life people^15^. For example, the “bald” attribute is under-represented in CelebA^24^, whilst it is a very common attribute among non-celebrities. At the training stage, imbalanced data induces AI models to pay more attention to learning the features of majority samples^27,28^. Consequently, there is a need for datasets covering more real-world settings and a wider range of facial appearances to achieve fair and non-discriminatory facial processing systems.

It is also important to highlight that private datasets from big internet giants such as Google and Facebook are up to $12\times$ larger than the largest public dataset (c.f. two last rows in Supplementary Table 1). The power that large companies have over large datasets favours the AI gap between industry and academia, and between tech giants and smaller companies. SMEs are particularly harmed by this gap, as most public facial datasets limit their use to “non-commercial research”. Nevertheless, there are many possible ambiguities in a “non-commercial” designation for a dataset. For example, it is unclear how non-profits and governments can use the dataset. It is also very difficult to trace whether a commercial model has been trained on non-commercial data and indeed, recently, evidence has been found on these kinds of malpractices^29^. Clarifying these legal uncertainties and designing contracts that would allow for different data sharing mechanisms between SMEs and large companies could eventually contribute to Trustworthy AI requirement (6) “societal well-being”.

Finally, the topic of facial datasets is directly related to requirement (3) “data governance”. In the last few years, some recommendations for documenting datasets have emerged^30^. They aim at providing standardized processes so that each dataset is accompanied with a datasheet that documents its motivation, composition, collection process, labeling scheme, conditions of distribution and maintenance, among others. These initiatives also promote requirements (7) “accountability” and (4) “transparency”, so that dataset users are aware of allowed uses and potential data biases. While these recommendations have started to be followed in other AI fields^31^, they have not been applied yet to facial processing.

### Towards context-aware evaluation strategies beyond accuracy-centred metrics

Traditional evaluation strategies centre around the idea of comparing facial processing systems’ outputs with manual annotations using a set of accuracy-related metrics. Most popular metrics reported in facial processing benchmarks include: overall accuracy, precision, recall, false positive rate, F1 score, confusion matrix, receiver operating characteristic curves, normalized mean error and FrÃƒÂ©chet inception distance. For a detailed description of these metrics and the computational tasks to which they are applied we refer the reader to Supplementary Fig. 2. Academic works mainly target the pursuit of these accuracy-centred metrics, which are nowadays almost saturated for most popular facial benchmarks^26^. However, such metrics are not always sufficient to indicate the system’s overall performance in real-world applications.

Let’s imagine a dummy gender classifier that always produces the output “man” regardless of the input facial image. If this system was to be validated on LFW^32^, acknowledged as the most widely used dataset in the field^33^, which contains 74% images labeled as “men” (c.f. Supplementary Table 1), its overall accuracy would be equal to 74% when in reality it would be misclassifying more than 50% of the population worldwide. Further, this system does not consider individuals that identify as non-binary in terms of gender, thus not reflecting existing gender diversity. Researchers should study accuracy metrics in context and elaborate on what the results imply. For instance, is a system with 99% accuracy better than one with 95%, if the latter favours gender fairness?

Another illustrative example is a face identification system in a crowded international scenario, such as an airport, with people coming from all over the world. On the one hand, the system has to be unbiased in demographic terms. On the other hand, when hundreds-to-thousands of faces have to be analysed in real-time, computational costs (i.e. high processing speeds) become critical even at the expense of tolerating some accuracy loss (e.g. at the expense of generating more false alerts in a targeted search of a missing child). Current facial processing benchmarks and competitions’ leaderboards simply list accuracy-centred metrics as raw numbers^34^ without elaborating on these other important issues.

The aforementioned examples have highlighted different evaluation challenges towards Trustworthy AI. First, while accuracy-centred metrics provide evidence on requirement (2) “technical robustness”, they might also make the user fall into over-reliance^35^ on the system and thus negatively impact requirement (1) “human agency and oversight”. Second, demographic-aware evaluations are needed to promote requirement (5) “diversity, non-discrimination and fairness”. Third, evaluating computational costs is not only important for assessing the real-time capabilities of a system but also its energy consumption, which potentially contributes to sustainable AI^36,37^ and requirement (6) “environmental well-being”. Thus, there is a need for more holistic and context-aware ways of evaluating facial processing systems, beyond the mere assessment of accuracy of individual computational tasks.

The U.S. National Institute of Standards and Technology (NIST) has begun to forge a path in this direction. It publicly reports a series of facial processing benchmarks, such as the Face Recognition Vendor Test (FRVT)^19^, which takes into account some additional evaluation factors, namely computational performance and demographic-awareness. Systems taking part in the competition are developed by leading commercial vendors worldwide and some research labs. However, strict submission policies (e.g. participants can only send one submission to the FRVT every four calendar months and evaluation datasets are not public) hinder researcher’s and practitioner’s free evaluation of their algorithms. Only recently, research works have started to address these additional aspects of evaluation in a more open manner.

Since the presentation of pioneering study on gender and racial biases in commercial facial categorisation in 2018^38^, an increasing -yet still very preliminary- effort is devoted to bias mitigation and the evaluation of fairness in facial processing systems. This effort comes mostly in the form of algorithms that can be used to mitigate bias either at the data level (e.g. by re-sampling training data to create a balanced dataset) or at the processing level (e.g. by penalizing the misclassification of minority groups during training). For a comprehensive review on such techniques we refer the reader to^39^. Demographic-aware protocols at the evaluation level are scarcer. The few existing ones include DemogPairs^27^ and the subsequent work Balanced Faces In the Wild^40^, which provide a facial dataset with varying ethnicity-gender verification pairs allowing to compare the gaps in accuracy between demographic groups. Similarly, Cross-Age Labeled Faces in the Wild^41^ proposes a protocol with varying age, and the FRUITS^26^ protocol goes a step further by taking into account all demographic variations (ethnicity, gender and age). Nevertheless, these protocols only target the face verification task.

Regarding facial processing contexts restricted by inference time, it is important to evaluate the trade-off between accuracy and real-time performance. It is common that academic works do not provide computational complexity metrics or that they give, at most, some overall timings achieved by the system on a particular hardware. More advanced initiatives are just starting to appear, motivated by the increasing use of facial processing in mobile and embedded devices with limited computational resources. For instance, the *lightweight face recognition challenge*^42^ constraints Floating Point Operations Per Second (FLOPs) and model size of submissions, and the FRUITS protocol restricts the verification of one image pair to 100, 500 and 1000 milliseconds^26^. However, with the recent emergence of 5G networks and edge computing^43^, companies are increasingly deploying distributed systems that allow for large-scale scenarios that seemed impossible until recently (e.g. analysing tens of video streams coming from different locations in a city, as in Supplementary Fig. 3). Thus, there is a need to design more sophisticated computational complexity benchmarks and simulations, considering factors such as video latency, the number of video streams a system is able to analyse in real-time and carbon footprint.

### The challenge of preserving privacy and security

Despite the benefits of distributed computing, there are increasing concerns with privacy and security, which are directly related to the requirements for Trustworthy AI (2) “safety” and (3) “privacy”. This refers particularly to facial and biometric data^44^. For example, it is common that face identification (FI) deployments send both extracted facial snapshots and biometric templates through a network (e.g. 5G or Internet) to a central station by means of secure connections (c.f. Supplementary Fig. 3). Other alternative designs are feasible such as sending only biometric information or, conversely, sending only facial images through the network and perform biometric template extraction in the central software. Each of these designs has different privacy and security needs which translates into different legal consequences.

Since very recently, the distributed computing paradigm has also started to be applied at AI systems’ training stage. Classic machine learning approaches require centralising training data in one single workstation and/or shared database. *Federated learning*^45^ is an emerging field aimed at collaboratively training an AI model by using parameters of locally trained models, keeping raw training data local (e.g. on local PCs, mobile or other connected devices). It is increasingly becoming a privacy-preserving approach of the utmost importance, specially in contexts such as healthcare where data confidentiality is strictly regulated. However, the potential of federated learning for facial processing applications is virtually unexplored. To the best of our knowledge, there is only one work to date making use of this technique to recognise facial expressions of pain^46^.

It is also interesting to note that some facial processing tasks can strengthen the security of others or, on the contrary, attack them. Pioneering works on facial attribute manipulation (FAM) have explored algorithmic solutions to generate recognizable biometric templates that can hide some of the private information (e.g. gender) present in facial images^47^. Further research on visual cryptography and biometric template protection^48^ to protect users’ privacy on facial images is essential. FAM has nevertheless negative dual uses, such as spoofing. Digital manipulation attacks can generate entirely or partially modified photorealistic faces in terms of expression, identity or facial attribute swaps. Other manipulation attacks introduce a small noise or perturbation in the input image, not perceptible to the eye, that for some reasons—that are not yet fully understood—breach most face identification and verification systems^49^. On the other hand, face spoofing detection (FSD) techniques aim at detecting these and other attacks. For instance, perturbation detection techniques have been recently proposed^50^. However, FAM is a rapidly growing research topic and new types of adversarial attacks are continuously appearing, challenging systems’ privacy and security.

### The need for more explainable facial processing systems

In machine learning we can distinguish between interpretable and black-box models^51^. Interpretable models are understandable either because they have a simple mathematical expression (e.g. linear models) or because their representation allows users to understand their behaviour (e.g. decision trees). Black-box models have complex mathematical expressions that do not possess a representation that can enable such an understanding. The increasing use of deep learning as a black-box approach has made facial processing systems lose explainability. This has negative implications for the trustworthiness of a system, more specifically for requirements (4) “transparency” and (1) “human oversight and agency”, as users should be given informed knowledge to comprehend the system and assess its outputs and decisions. The way to tackle this problem is to equip black-box models with some *explainability* mechanisms (e.g. visualizations or approximations to interpretable models)^52^.

Only a few early attempts have been made towards explainable facial processing systems. For example, an “explainable face recognition” (XFR) protocol is presented in^53^ to generate an attention map highlighting the facial regions that are responsible for a matching. The work in^54^ uses similar representations to explain how a deep neural network distinguishes facial expressions of pain from facial expressions of happiness and disgust. Interestingly, the study in^55^ explores the opposite direction: how detected facial Action Units can be used to adapt the explanations provided to the user in a gaming context. Nevertheless, the design of explainability mechanisms is still widely under-explored in the field. A key challenge is the lack of ground truth to compare and quantify explainable results across models. Further, there is no consensus in the research community on how to assess and measure the quality of an explanation. The few works addressing the topic agree that not all applications and stakeholders have the same interpretability needs, and therefore interpretability assessment should be a contextualized process taking into account the application domain and the use case at hand^56,57^.

### Public perception of facial processing

In recent years, multiple incidents with facial processing technologies causing racist and other discriminatory outcomes^58^, disinformation^59^ and privacy invasion^60^ have painted a highly negative picture of the entire research field. This has been reinforced by some controversial applications outlined in media that have become widely known by the general public^61^.

Several studies have analyzed public perception of facial processing, with a focus on identification scenarios. A survey with 4,109 adults in the UK^62^ found out that, even if the awareness of facial recognition technology is high, public knowledge is still low, e.g., on where facial recognition is used, its accuracy and limitations. This finding calls for efforts in public outreach and education. In addition, the study showed that although people have certain fears and there is no unconditional support for police usage, many respondents felt reassured with consent as an important safeguard, and support the use of the technology when there is a demonstrable public benefit. Finally, the survey found that respondents do not trust the private sector, support companies pausing sales and expect governments to place limits. This tension between privacy concerns and support for a more effective security and law enforcement is confirmed by another survey with 2,291 persons in Australia^63^, endorsing as well the need for public education, consent, rigorous testing and meaningful regulation.

Recent research also shows that public perception highly relates to cultural background. A study on public attitudes towards face identification in criminal justice in USA, China, UK and Australia^64^ found, for instance, that USA respondents are more accepting of the tracking of citizens and private use of technology; they are less trusting of the police than people in the UK and Australia, and that Chinese and Australian respondents think the technology is more accurate than people from UK. This illustrates the need for culture-aware approaches for the development of technologies and use cases. Finally, these studies reveal the need for public outreach on a wide range of application scenarios of facial processing, especially those for social good.

## Summary and conclusions

In this work, we have identified 12 facial processing computational tasks addressing different goals, from the detection of faces in still images to the recognition of emotional expression, lip reading or the manipulation of facial attributes in videos. Some of this research is already integrated in different real-world scenarios with different levels of market penetration and social impact. We have collected 60 of such scenarios, quantified the number of companies having related products in the market and assessed their risk level according to the European AI Act.

We found that there are many “high-risk” applications in the market, even though some challenges are still to be solved to ensure that these systems are developed and evaluated in a trustworthy way according to the use case they will be used for, as required by the AI Act. Table 5 summarises identified challenges in the context of the 7 requirements for Trustworthy AI.

**Table 5:** Summary of the main challenges and research needs towards trustworthy facial processing identified in this work.

Requirement for	Main challenges and research needs towards trustworthy facial processing	
Trustworthy AI	Challenge	Research needs
1. Human agency and oversight	The **explainability **of current facial processing systems is limited.	Investigate on new ways of providing users with the most **meaningful information and interaction mechanisms** to understand, assess and potentially override systems’ outputs and decisions.
	Too general accuracy-centered metrics are provided to the users as a proxy to systems’ performance, which might cause **over-reliance on the system and disinformation**.	Design **more informative metrics** of a system’s performance which take into account its context of use and intended purpose.
2. Technical robustness and safety	Existing **facial datasets are highly noisy** in terms of data incorrectly labeled, with consequent high detrimental impact on models’ robustness.	Improve the quality and annotations of existing facial datasets, in the pursuit of AI Act’s **“error free”** objective.
	Current **evaluation benchmarks and leaderboards are limited** to rank facial processing models using a set of accuracy-centred metrics on a particular dataset.	Research on **holistic evaluations** beyond accuracy-centred metrics (e.g. measuring the real-time capabilities of a system, its demographic biases, etc.), more informative about the robustness of the system in its operational setting.
	Facial processing systems are **more and more distributed and large scale**, which raises increasing security concerns.	Increase **resilience to attack and security in connections** involving sending facial images and biometric templates.
3. Privacy and data governance	Existing **facial datasets are not well-documented**. Important information such as dataset composition, collection and annotation process, labeling schemes, potential data biases, allowed uses or conditions of distribution and maintenance is usually missing.	Develop **open facial datasets, documented in a comprehensive, standardised and auditable way**.
	The training of facial processing systems requires compiling and **centralising training facial data in one single machine or shared database**, which causes privacy and confidentiality issues.	Investigate on **federated learning techniques** to train facial processing systems in a decentralised way, without the need of moving data from their local origin.
	New forms of **adversarial attacks** for the purpose of spoofing facial processing systems are continuously appearing (e.g. digital manipulation attacks, perturbation attacks).	Research on **techniques to protect users’ privacy** on facial images (e.g. visual cryptography, perturbation detection, biometric template protection).
4. Transparency	The** lack of clear and transparent documentation on facial datasets’ limitations** has a strong negative impact on facial processing systems’ training, testing and evaluation.	Dataset creators to **formally document datasets’ limitations** (e.g. data biases, annotation quality).
	Current facial processing systems are commonly used as **black boxes**.	Investigate **specific explainability and interpretability techniques for facial processing**, enabling the understanding of how the systems make decisions.
5. Diversity, non-discrimination and fairness	Facial datasets are **extremely imbalanced with regard to demographic factors** such as age, gender and ethnicity. Also, as a consequence of the way they are built (massive web crawling in search of celebrity images), they are not representative of real-world people.	Create **demographically-diverse and -balanced datasets** representing real-world people.
	Most popular evaluation benchmarks do not provide protocols to **assess the behaviour of the system in demographically-diverse contexts**.	Create **demographic-aware evaluation protocols** allowing to assess gaps in accuracy between demographic groups.
6. Societal and environmental well-being	Facial processing systems are becoming increasingly distributed and computationally complex, and their deployment might entail a **high energy consumption and carbon footprint**.	Research on **environmental-aware facial processing**.
	Largest facial **datasets are only in the hands of big Internet giants and some governments**.	In order to give the opportunity to smaller companies and institutions to create competitive and innovative facial processing products, establish **data sharing initiatives** between SMEs, governments, non-profits and large companies.
	**Public knowledge and trust** in facial processing is still low, and there is no unconditional support for good uses of the technology.	**Outreach and educate** the general public about the wide range of uses of facial processing, their benefits, risks and limitations.
7. Accountability	There are **legal ambiguities on the allowed uses of existing public facial datasets** outside academia, e.g., by third parties such as non-profits, governments and companies.	State the allowed uses and conditions of distribution **formally and in a standardised way** in the documentation accompanying a dataset.

We have also reviewed a series of studies showing how public perception of facial processing depends on culture and reflects the tension between privacy concerns and support for applications for the public good. Those studies support the need for education, rigorous evaluation, and regulatory limits. The fact that their focus is on facial identification may indicate that some of the use cases identified in this study, particularly those having a strong positive impact, are not so well-known. This includes the use of facial technologies for the prevention of Covid-19 spread, for accident prevention, to improve accessibility for the blind, to build social robots as companions for the elderly or to assess pain in patients with communication problems. We found that SMEs have a key role in pushing towards those positive social impact applications but they also face difficult challenges such as limited access to facial datasets and the negative public perception.

Policies addressing facial processing should balance opportunities vs risks, favour the market integration of innovative SMEs, and ensure that the technology is evaluated and exploited in a trustworthy way and in scenarios with a positive social impact. The media, policy makers^65^, researchers, scientists and vendors should all take the responsibility and commitment to promote trustworthy facial processing.

## Supplementary Information

Supplementary Information.

## Data Availability

The data generated and analysed during the current study are available from the corresponding author on reasonable request.
