# Supplementary material for: The landscape of facial processing applications in the context of the European AI Act and the development of trustworthy systems
Source: Sci Rep. 2022 Jun 23;12:10688. doi: 10.1038/s41598-022-14981-6 (PMC9223252; doi:10.1038/s41598-022-14981-6)
Supplement: Supplementary file 1 — Supplementary Information. [file 41598_2022_14981_MOESM1_ESM.pdf]

Supplementary Information for *The landscape of facial processing applications in the context of the European AI Act and the development of trustworthy systems*

Isabelle Hupont[1*,], Songül Tolan[1,**], Hatice Gunes[2], and Emilia Gómez[1,**]**

[1]Joint Research Centre, European Commission, Seville, Spain
[2]Department of Computer Science and Technology, University of Cambridge, United Kingdom
[*]Corresponding author: isabelle.hupont-torres@ec.europa.eu
[**]The opinions expressed are those of the author(s) only and should not be considered as representative of the European Commission's official position.

SUMMARY

This supplementary information file complements the main manuscript *"The landscape of facial processing applications in the context of the European AI Act and the development of trustworthy systems"* by providing additional figures, tables and results.

Please note that we refer to the different facial processing tasks identified in the main manuscript either by using their full names or their acronym, as depicted in Supplementary Figure 1.

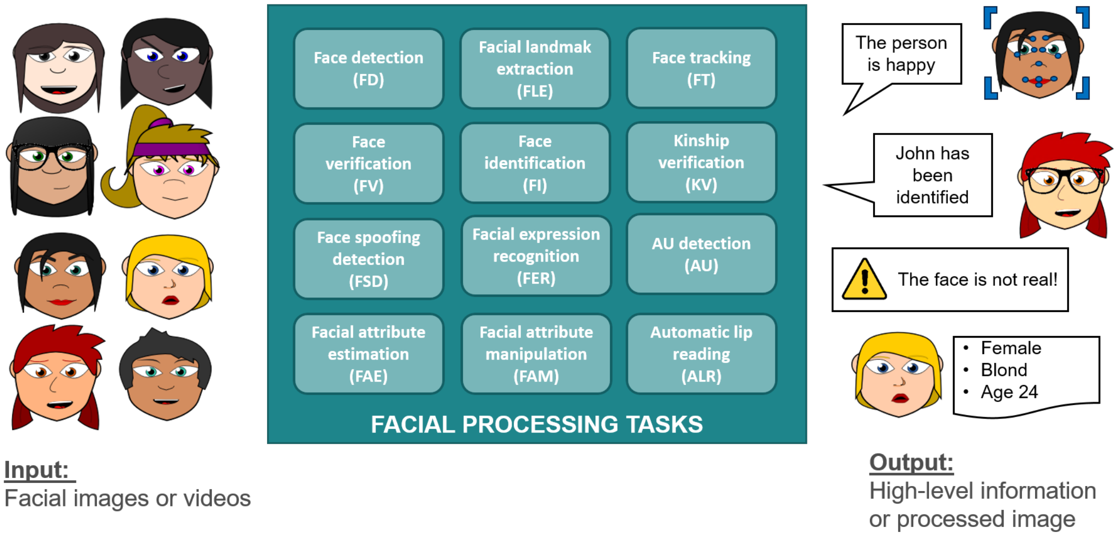

Supplementary Figure 1. Facial processing tasks covered in this work and their corresponding acronyms (in brackets). Face drawings are courtesy of *Pixabay* (https://pixabay.com).

Facial datasets analysis

Supplementary Table 1 provides the main characteristics of most popular and/or state-of-the-art facial datasets, including the facial processing tasks for which they are used. Top rows are datasets publicly released, while the last two are private and owned by the Internet giants Google and Facebook.

**Dataset**	**Main use**	**#Faces**	**#IDs**	**Demographic diversity**			**Annotations**				**Cond.**	**Noise**
				Ethnics (A, B, W)	Age	Gender	Expressions	Demog.	Land.	Attr.		
LFW[1]	FI, FV	13K	5.7K	6%, 9%, 85%	20-75	26% F	No	No	No	No	W	<2%
IMDB-Face[2]	FI, FV	1.7M	59K	23%, 12%, 65%	10-70	45% F	No	G, A	No	No	W	<2%
DemogPairs[3]	FI, FV	10.8K	600	33%, 33%, 33%	15-75	50% F	No	G, E	No	No	W	C
MS-Celeb-1M[4]	FI, FV	10M	100K	70% W	20-80	80% F	No	No	No	No	W	>50%
WebFaces42M[5]	FI, FV, FAE, FAM	42M	2M	20%, 10%, 70%	20-80	38% F	No	Ag, E, G	No	4	W, L	<10%
DISFA[6]	AU, FER, FLE	130K	27	11%, 4%, 85%	18-50	44% F	12 AU	No	Yes	No	L	C
BP4D[7]	AU, FER, FLE	140K	41	27%, 14%, 59%	18-29	56% F	8 BE, 12 AU	No	Yes	No	L	C
AffectNet[8]	FER, FAE, FLE	450K	NA	NA	15-60	51% F	7 BE, VA	No	Yes	No	W	C
CelebA[9]	FAE, FAM, FI, FV, FLE	203K	10.2K	NA	7% Y	46% F	Smile	Ag, G	Yes	37	W	<2%
LFWA+[10]	FAE, FAM	13.2K	5.7K	6%, 9%, 85%	20-75	26% F	Smile	Ag, E, G	Yes	49	W	C
WIDER[11]	FD	394K	NA	NA	NA	NA	No	No	No	No	W	C
MALF[12]	FD	11.9K	NA	NA	NA	8% F	EE	G	No	3	W	C
CelebA-S[13]	FSD, FI, FV	625K	10.2K	NA	7% Y	46% F	Smile	Ag, G	Yes	37, S	W	<2%
FIW[14]	KV	3.7K	10.7K	NA	NA	NA	No	No	No	KR	W	C
LRS2-BBC*[15]	ALR	250h	1K	NA	NA	NA	50K words	No	No	No	W	C
Facebook[16]	FI, FV	500M	10M	NA	NA	NA	NA	NA	NA	NA	W	NA
Google[17]	FI, FV	200M	8M	NA	NA	NA	NA	NA	NA	NA	W	NA

* LRS2-BBC is a video dataset, thus we provide the total number of recording hours instead of *#faces*. Also, annotations are given in the form of spoken words (in English) which convey different facial expressions or visemes.

Supplementary Table 1. Main characteristics of most popular and largest datasets for facial processing, as provided by their authors or in subsequent papers. List of abbreviations: A, B and W represent respectively the percentage of Asian, Black and White people in the dataset; Y = young; F = female; BE = basic emotion; VA = valence-arousal; EE = exaggerated expression; G = gender; Ag = age; E = ethnicity; S = type of spoofing attack; KR = kin relationship; W = wild (unconstrained); L = lab (controlled); C = clean; NA = information not provided. Note that We do not include datasets for face tracking (FT), as general multiple object tracking (MOT) datasets are commonly used instead for this purpose[18].

Most popular evaluation metrics in facial processing

Supplementary Figure 2 compiles and illustrates most widely used evaluation metrics that can be found in facial processing benchmarks and leaderboards, and indicates the computational tasks to which they are applied. As it can be observed, these metrics are mostly accucary-based and include: overall accuracy, confusion matrix, precision, recall or True Positive Rate (TPR), False Positive Rate (FPR), F1 score, Receiver Operating Characteristic (ROC) curves and Normalized Mean Error (NME). In the case of face tracking (FT), performance is further evaluated over time in terms of tracking trajectory fragmentations and identity switches, but still following the classic accuracy concept of error assessment with respect to manual ground truth. Facial attribute manipulation (FAM) is the only exception to the accuracy concept as the objective in this case is the generation of credible (i.e. photorealistic) imagery that falls within the distribution of real images. This is usually assessed using the Fréchet Inception Distance (FID)[19].

Metric	Description	Illustration	Tasks reporting the metric
**Accuracy**	Measure of correct classified samples against the total number of test samples.	$Accuracy = \frac{True\ Positive + True\ Negative}{Total(Positive + Negative)}$	FD, FT, FV, FI, KV, FER, AU, FSD, FAE, ALR, FAE, FAM
**Confusion matrix**	Given a classification problem with N different classes, the confusion matrix is a NxN matrix, whose elements a_{ij} represent the amount of elements of class j that have been predicted to belong to class i.	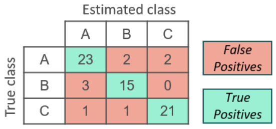	FER, AU, FSD, ALR
**Precision**	Measures a model's ability to not label a sample as positive if it is negative.	$Precision = \frac{True\ Positive}{True\ Positive + False\ Positive)}$	FD, FT, FER, AU, FSD
**TPR (recall or sensitivity)**	The True Positive Rate (TPR), also known as recall or sensitivity, represents the fraction of positive samples that were correctly labelled as positive.	$TPR = \frac{True\ Positive}{True\ Positive + False\ Negative}$	FD, FT, FV, FI, KV, FER, AU, FSD
**FPR**	The False Positive Rate (FPR) represents the fraction of negative samples that were incorrectly labelled as positive.	$\mathrm{FPR} = \frac{False\ Positive}{True\ Positive + False\ Negative}$	FD, FT, FV, FI, KV, FSD
**F1 score**	It is the weighted harmonic mean of precision and recall (F1=1 is best).	$\mathrm{F1} = 2\,x\,\frac{Precision\ x\ Recall}{Precision + Recall}$	FD, FT, FER, AU, FSD
**ROC Curve**	The Receiver Operating Characteristic (ROC) curve is a graphic metric, showing FPR vs TPR. It illustrates the discrimination capability of the model at various discrimination thresholds (green arrow).	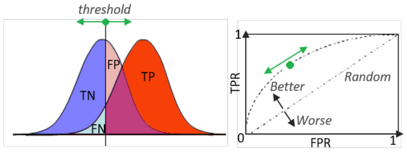	FD, FV, FI, KV, FSD
**AUC**	It measures the area underneath the ROC curve. The larger the AUC area, the better (AUC=1 is best).		
**NME**	The Normalised Mean Error (NME) computes the normalised mean distance between predicted and ground truth N facial landmarks' positions <x_i,y_i>. Normalisation factor *d* is generally the inter-ocular distance.	$NME = \frac{1}{N}\sum_{i=1}^{N}\frac{\sqrt{\Delta x_i^2 + \Delta y_i^2}}{d}$	FLE
**Fragmentations and ID-switches**	Metrics related to tracking. A fragmentation happens when the ground truth (GT) trajectory is lost, while an ID-switch is produced when the tracker switches the correct face (identity or ID) to another one.	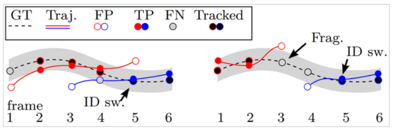	FT
**FID**	Fréchet Inception Distance (FID) calculates the distance between the distribution of feature vectors of generated images (P_g) and the distribution of real images (P_r).	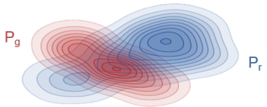	FAM

Supplementary Figure 2. Compilation of most commonly reported metrics in facial processing benchmarks and leaderboards. The illustration of fragmentation and ID-switches is adapted from[20].

Implementation example: unconstrained large-scale face identification at the edge

Supplementary Figure 3 illustrates a possible edge computing architecture which could be used to deploy a remote large-scale face identification system, such as the one considered in use case BI8. It follows a distributed computing paradigm, where a scalable number of edge nodes installed on-premise extract facial snapshots and corresponding biometric templates from live video streams. A central software installed at a control room (e.g. a server located at the Police office) orchestrates the information coming from all edge nodes and performs facial matching. Generated identification alarms can be finally sent to on-premise law enforcement agents (e.g. to their smartphones or tablets) so that they undertake necessary actions.

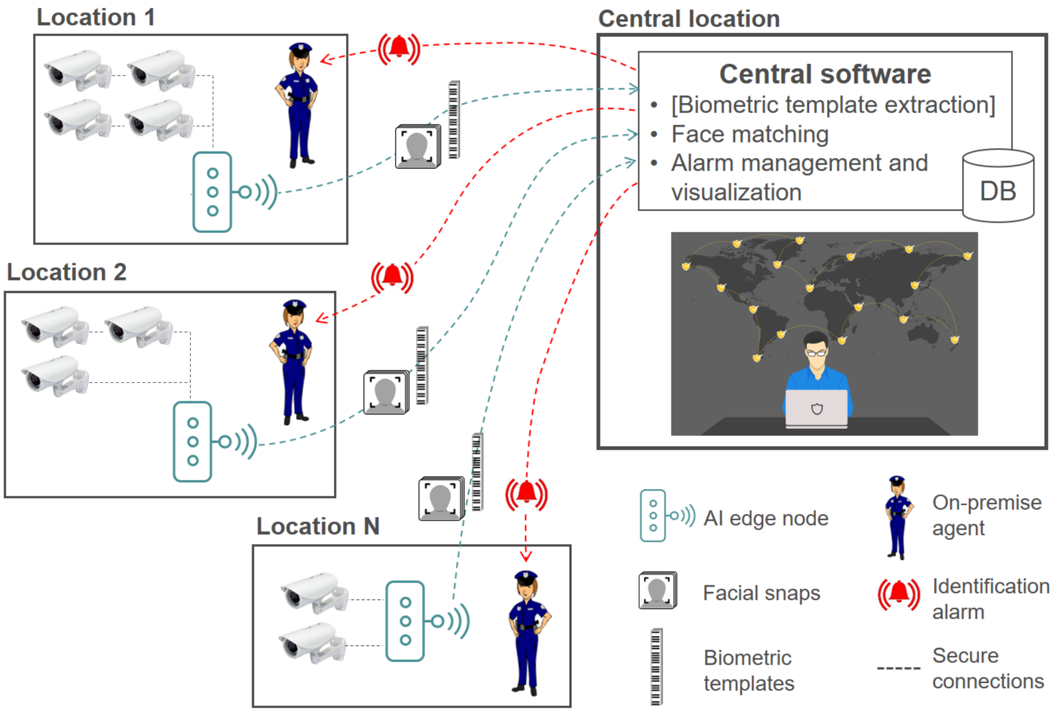

Supplementary Figure 3. Example of edge computing architecture for remote face identification from multiple geographic locations which could be use for the implementation of use case BI8 *"Unconstrained face identification"*. Police drawings are courtesy of *Pixabay* (https://pixabay.com).

Detailed information on identified facial processing applications

This supplementary section provides further information on the 60 use cases presented in the main paper. More particularly, each identified application is described in detail and accompanied by reference papers, corresponding facial processing tasks and key companies having products or having participated in related real deployments. Applications are divided into four separate tables, depending on the type of AI system they implement according to the AI Act:

- **Biometric Categorisation (BC).** Supplementary Table 2 presents 7 use cases using categorisation systems from facial images.
- **Biometric Identification (BI).** This category includes applications implementing "remote", "non-remote", "real-time" and "post" BI systems. A total of 20 facial BI use cases are presented in Supplementary Table 3.
- **Emotion Recognition (ER).** A total of 18 facial emotion recognition applications are enumerated in Supplementary Table 4.
- **Other (OT).** Supplementary Table 6 depicts 15 other facial analysis applications that do not belong to the categories above.

Note that, for the sake of clarity, a 3-letter code has been used in the tables to refer to companies. The correspondence between the name of each company and its code can be found later in Section *List of key companies on facial processing*, together with other relevant information about the company, namely its headquarters country, website URL and size (SME or large).

#	Application	Description	Tasks	Areas	Companies
BC1	Demographic analysis	Extraction and aggregation of demographic statistics (age, gender, race) to obtain customers or visitors profiles (e.g. percent of young females visiting a shop or museum during the weekend). The cameras can be located on the counters of a store, in a storefront (e.g. embedded in mannequins), at the entrance of a museum, etc.[21,22]	FD + FT + FAE	BIC, MKT, TOU	ISS, HER, VIV, UNI, SCV, ETI, QUA, SIG, HBI, MEG, COG, VIX, ROC, TOS, GOR, RAI, EVO, AGV, VOC, VAT, KED, RNE, 3DI, AMA, ALM, FEL, OMR, PAC, VTE, CLA
BC2	Person search by facial appearance	From a large collection of video footage or images, automatic search for persons fulfilling a certain facial description (e.g. asian female wearing hat or middle-age bald white men with glasses). These tools are used, for instance, by broadcasters to retrieve specific multimedia contents, or by LE bodies to search for specific person profiles as described by witnesses.[23–26]	FD (+FT) + FAE	BIC, LE, VSU, ENT	BFC, KED
BC3	Face mask detection	Detect whether people are wearing or not medical masks (e.g. in a restaurant, shop, train, operating room, public spaces, etc.). Law enforcement bodies could take actions against persons not wearing a mask (e.g. fining them).[27–29]	FD (+FT) + FAE	BIC, CLI, LE, VSU, TOU, MKT, TRA	ISS, HER, IPS, BFC, INN, SIG, DBA, CYB, VIX, ROC, PAR, GOR, TFA, CBL, RNE, DON, FEL, ID3, HID
BC4	Detection of personal attributes	Guess a person's personal attributes such as attractiveness, sexual orientation, political orientation, potential criminality or psychopathy traits from facial images.[30–34]	FAE	BIC, EDU, EMP, SER, JUST, MIG, SOC, FIN	-
BC5	Personalisation of advertising content	Automatic adaptation of advertisements depending on the customer profile (age, gender, ethnicity, etc.) to engage consumer and drive sales.[35–37]	FD + FAE	BIC, MKT	SCV, QUA, HER, FFR
BC6	Verification for age-restricted goods	Facial age estimation, e.g., in electronic vending machines (to not sell alcohol to minors) or internet content (to block inappropriate adult content, gambling or online abuse).[38–40]	FD + FAE	BIC, MKT, ENT	SIG, CIV, IDE
BC7	Clinical syndrome assessment†	Automatic assessment of autism, schizophrenia, psychosomatic disorders, down syndrome and other syndromes based on facial analysis.[41–44]	FD (+FT) + AU/FAE/FER	BIC, CLI	BSA

Supplementary Table 2. List of 7 facial analysis applications under the category of Biometric Categorisation (BC). For the application marked with †, the AI system could potentially be a safety component or part of a medical device.

#	Application	Description	Tasks	Areas	Companies
BI1	Access control*	Allow or prevent unauthorized persons to access critical infrastructures (e.g. official buildings) or other facilities (e.g. schools, workplace, hospitals, logistic centres) by means of face identification. Can also be used to prevent blacklisted people to enter casinos (e.g. gambling addicts), sport stadiums (hooligans), banks, means of transportation (planes, trains), events (e.g. banned fans), etc. Anti-spoofing mechanisms should be included to avoid attacks.[45–47]	FD + FI (+FSD)	BIC, MCI, VSU, TRA, ENT, CLI, EDU, TOU, EMP, LE, FIN, IND	ISS, HER, IDE, HIK, NEC, AVI, INN, INN, FFR, CLA, PAN, HBI, SEN, AYO, CYB, MEG, CLO, COG, THA, HIS, VIX, ROC, PAR, TOS, GOR, TFA, PSO, RAI, EVO, TEC, AGV, CAM, CBL, KED, RNE, DON, REN, IRE, 3DI, CLR, CCO, FEL, HOO, YIT, ITL, ZKT, VTE, CLA, AWA, ID3, BID, FUL, HID
BI2	Access control with masks*	Identify faces for access control without the need for people to remove their medical masks. This may reduce the risk of contagion in pandemic context.[48–50]	FD (+FAM) + FI (+FSD)	BIC, MCI, VSU, TRA, ENT, CLI, EDU, TOU, EMP, LE, FIN, IND	SEN, HER, HAN, CYB, PAR, GOR, CBL, RNE, DON, BID
BI3	Border control*	Verify a person's actual face against the face stored in his/her travel document (e.g. Automatic Border Control "ABC" gates).[51–53]	FD + FV + FSD	BIC, MIG, LE	THA, VBX, SIT, PAN, AYO, MEG, COG, HIS, TOS, DER, PAG, ITL, VTE, AWA, TEB, ID3
BI4	Banking authentication*	Operate in ATM or make transactions with the mobile phone using FV, without the need of a card or PIN. It should include spoofing detection mechanisms.[54–56]	FD + FV + FSD	BIC, FIN, MKT	FPH, ISS, HIK, IDE, MEG, CLO, VIX, ROC, PAR, TOS, TFA, CBL, DER, REN, CLR, KAI, SSY, AMA, YIT, ITL, AWA, DAO, ID3, HID
BI5	Sousveillance	“Sousveillance” denotes bringing the video surveillance camera down to human level, e.g., by using videos from bodycams worn by LE bodies to identify blacklisted persons while walking in the streets or critical infrastructures. Identification alarms might be sent to users through diverse channels (audio, wearables, smartphones, etc.).[57,58]	FD (+FT) + FI	BIC, LE, VSU, MCI	WOL, MAN, DBA, COG, AVI, ROC, MOT, CAM, 3DI, LLV
BI6	Devices, machines and data unlocking*	Unlock devices (e.g. smartphone, PC, smart home devices), machines (e.g. vehicles, industrial machines, security lockers) or information (e.g. access to medical apps, databases, images, etc.) using the face.[59–61]	FD + FV + FSD	BIC, ENT, IND, TRA, CLI, MCI	APL, GOO, IDE, MIC, FFR, HBI, SEN, MEG, COG, ROC, TOS, HUA, MIC, GOR, 3DI, SSY, ITL, ZKT, VTE, AWA, ANI, DAO, ID3, BID, FUL, KLE, HID
BI7	Face authentication for e-Government*	Carry out administrative procedures (e.g. voting, tax declaration, access to census, employment history, medical records, certificates, etc.) through connected devices using face authentication. These procedures can be done at home with a smartphone, from a specific administrative booth, etc.[62–65]	FD + FV	BIC, SER, JUS, POL, EMP, CLI	Alicem (French Gov.), THA, PAR, DER, CLR, DAO, TEB
BI8	Unconstrained face identification	Real-time FI in moderate to densely crowded places (e.g. public spaces, sport stadiums, train stations, malls, airports, etc.), generally using a multi-camera system, to search for blacklisted people or missing persons. This application might cover up to very large geographically extended scenarios, such as a smart city.[66–68]	FD (+FT) + FI	BIC, LE, VSU, MIG, MCI	HER, NEC, AVI, SEN, INN, FFR, EYD, QUA, PAN, NTL, DBA, NEU, HBI, VLA, DEG, AYO, MEG, COG, THA, VIX, XFA, ROC, PAR, MOT, TOS, GOR, TFA, PSO, EVO, TEC, AGV, CAM, VOC, CBL, KED, RNE, REN, IRE, 3DI, AMA, YIT, ZKT, VTE, CLA, ANI, SVI, FUL
BI9	Person re-identification	Match people across non-overlapping camera views in a multi-camera system. It allows person tracking at a large scale to reconstruct trajectories (e.g. in a map).[69–73]	FD + FT + FI	BIC, LE, VSU, MCI	NEC, HER, XFA, KED, REN, YIT
BI10	Person search by identity**	Forensic search for a specific person (e.g. a missing child, a criminal, etc.) from a large collection of video footage based on existing reference facial image(s) of the subject. It can also be used for searching specific multimedia contents (e.g. interviews where a given actor appears).[74–76]	FD (+FT) + FV	BIC, LE, VSU, ENT	HER, NEC, AVI, INN, ETI, BFC, FFR, CLA, PAN, HBI, CYB, MEG, COG, VIX, ROC, MOT, TOS, EVO, TEC, AGV, CAM, VOC, VAT, KED, IRE, KAI, AMA, YIT, IFL, AWA, ANI
BI11	Contact tracing**	Identify where (e.g. infected or quarantined people) have been over time and how, when and where they have come in contact with one another.[77,78]	FD + FT + FI	BIC, LE, CLI	AVI, IPS, BFC, VIX
BI12	Person tracking from drones	Identify a target person (e.g. blacklisted or missing person) using imagery captured from drone-mounted sensors, and track this target person.[79–82]	FD + FT + FI	BIC, LE, VSU	TFA, AVI
BI13	Perimeter protection*	Protect sterile zones, critical infrastructure, high-risk sites, commercial, residential sites, retail spaces, or any other urban or remote location from unwanted intruders (allowing only authorised persons to be present).[83]	FD + FT + FI	BIC, LE, VSU, MCI	HIK, MIL, IPS, ETI, FFR, PAN, AGV, IRE
BI14	Control of attendance*	Check students, employees, registered persons' attendance to courses, exams, workplace or events.[84–86]	FD + FV/FI	BIC, EMP, EDU	JIB, HER, SEN, HIK, IPS, NTL, NEU, AYO, MEG, CLO, IDE, SCV, GOR, PSO, EVO, AGV, SAI, CCO, AMA, RES, PRU, PCT, HLK, EXA, AWA, DAO
BI15	VIP recognition	Let staff know when a VIP customer has entered a shop, restaurant, hotel, casino or building (e.g. to greet them by their name, offer special products, discounts, etc.). It might also include the identification of returning customers.	FD (+FT) + FI	BIC, MKT, ENT, TOU, FIN	ISS, HER, SCV, FFR, QUA, CLA, NTL, CYB, COG, VIX, ROC, TFA, PSO, AGV, REN
BI16	Face tagging in personal pictures and videos**	Automatic tagging of own and "friend" faces in stored personal images/videos (e.g. in a smartphone or in a social network profile). It might be used to group pictures into "albums" depending on the persons appearing in them.[87–90]	FD + FI	BIC, ENT	FBK, APL, MIC, SAM, SEN, MEG, COG, KAI, GOO, AMA, OMR, PAC
BI17	Assistance for people with visual impairments	Help individuals with visual impairments recognize other people. The system might also inform about people's emotional state. This application enables visually impaired to have more awareness of, and interactions with, other people in their surroundings. These systems are sometimes embedded in a wearable device (e.g. head mounted) to notify users about detected persons while walking.[91–98]	FD (+FT) + FI (+FER)	BIC, SOC, CLI	FBK
BI18	Person search in social networks**	Search for a given face (image reference) in social networks and collect all the images related to that person, plus information on who he/she likely is.[31,99–101]	FD + FV	BIC, LE, MKT, EMP, SER, POL	CLE
BI19	Mobile surveillance robots	Mobile surveillance robots that operate indoor (e.g. in a house, office, critical infrastructure, factory) with FI and FT capabilities. For instance, this kind of robots may recognize the members of a family or a company's staff, warn when intruders appear and follow them.[102–104]	FD (+FT) + FI	BIC, VSU, LE, IND, MCI	AIM, BFR, AMA
BI20	Product personalisation*	Identify the user's face and personalise a product according to her/his preferred configuration. E.g. in the case of a car, adjust automatically the seat, mirror, light, temperature, audio; in the case of a software, configure it with the user's preferred layout; in the case of a domotic system, adjust the home environment according to a family member's favorite presets.[105–107]	FD (+FT) + FI	BIC, ENT, TRA, MKT	VAL, GOO, ROC, KIA, VTE

Supplementary Table 3. List of 20 facial analysis applications under the category of Biometric Identification (BI). Applications marked with * and ** indicate “non-remote” and “post” BI, respectively.

#	Application	Description	Tasks	Areas	Companies
ER1	Smile detection	A smile detection algorithm automatically triggers the camera (e.g. smartphone camera, digital camera, photo booths, etc.) when all the persons facing it are smiling.[108–111]	FD + FAE/FER/AU	ENT	CAN, SON, KOD, OLY, APL, SAM, NIK, XIA
ER2	In-lab user experience testing	Facial emotion analysis for gauging results of preference for a product (physical product, software, video game, etc.) while the user is interacting with it. It might include the analysis of videos from focus groups or advertisement/movie watching.[112–116]	FD + FER (+AU)	MKT, ENT	AFF, UNI, NOL, VIV, IMO, DIS, KAI, OMR, REY, EMO, SEC, TAW, VTE, NVI, ETT
ER3	Crowdsourced user experience testing	Large-scale web-based crowdsourcing of people facial reactions while watching an advertisement or media content.[117–119]	FD + FER (+AU)	MKT, ENT	AFF, IMO, OXA, REY, TAW, ETT, ELH
ER4	Measure of consumer satisfaction	Extract and aggregate facial reactions (e.g. expressions, emotions, valence, arousal) to obtain customers, patients or visitors degree of satisfaction. It can be used for customers visiting a shop, patients in a hospital, visitors of a museum, etc.[120–122]	FD (+FT) + FER (+AU)	MKT, ENT, TOU, CLI	UNI, OXA, KAI, OMR, WSE, IFL, ETT
ER5	Student proctoring and tutoring	Capture a student's emotions (e.g. frustration, boredom) in a e-learning application, in order to warn for supplemental explanation or switch the way to enhance the effectiveness of education. Facial analysis might also allow to flag events during examination (e.g. to detect cheating or other suspicious behaviour).[123–126]	FD + FER (+AU)	EDU	OXA, RES, PRU, PCT, HLK, EXA, FSA
ER6	Job interviews	Assess candidates' emotions, mental states and personality traits during job interviews.[127–129]	FD + AU (+FER)	EMP	HRV, EMO, RET
ER7	Audience affective monitoring	Monitor audience facial reactions and focus of attention. E.g., dynamically spotlight most expressive reactions and distracted persons during a performance, concert, lecture, political plenary meeting, etc.[130–133]	FD + FER (+AU)	ENT, POL, EDU, CUL	MIC, SIG, KAI, REY, TAW
ER8	Emotional gaming experience	Improve gaming experience by appropriately responding to user facial expressions (e.g. boredom, frustration, enjoyment, engagement, etc).[134–136]	FD + FER (+AU)	ENT	-
ER9	Interactive emotional art	Adapt digital art contents to the viewer's facial emotions. E.g. emotions module the colours of a painting, the text of a poem, the contents of a video or the tempo of a piece of music.[137–139]	FD (+FT) + FER	ENT, CUL	CCH, REY, PAN, VTE, MCA
ER10	Emotional recommendation system	Music, movies, art or product recommendation system based on detected user facial emotions.[140–143]	FD (+FT) + FER	ENT, CUL, MKT	-
ER11	Driver monitoring and warning†	Monitor driver's face to detect fatigue, stress, aggresivity, distraction or drowsiness, warning her/him in real-time through alerts and prompts. It can also apply to monitoring pilots during a flight.[144–147]	FD + FER (+AU)	TRA	AFF, SEE, LEX, GMO, FOR, VAL, HON, HBI, SEN, COG, EYA, VTE, NVI
ER12	Driver monitoring for autonomous vehicles†	Same as previous, but the vehicle might take autonomous decisions (e.g. taking partial or full control of a car in case of drowsiness, adjust the interior vehicle's cabin to driver's mood to calm him/her down, etc.).[148, 149]	FD + FER (+AU)	TRA	SEE, VAL, KIA, HYU, EYA
ER13	Affective interaction with agents	Provide social robots, embodied virtual agents or personal assistants with FER capabilities to engage and interact emotionally with humans.[150–155]	FD + FER (+AU)	ENT, MKT	SOF, FUR, ICU, JBO, HON, ROB, FUJ, IMC, BFR, ZOR, EMB, ANK, EMT, BSA, IFL
ER14	Affective robots as companions for elder people†	Companion robots with FER capabilities to assist elder people, reduce their stress and depression, enhance social interaction and elicit emotional responses. This kind of robots have demonstrated to address the difficult challenges of loneliness, isolation and dementia.[156–159]	FD + FER (+AU)	CLI, SOC	ZOR, BFR, SOF, EPR, SON, FUJ, PRO
ER15	Social robots for children with autism†	Social robots with affective capabilities to support the academic and social development of children with autism. They might be used to train social skills (e.g. decoding/encoding facial expressions) and as mediators between autistic children and others, favoring a greater social inclusion.[160–164]	FD + FER (+AU)	CLI, SOC, EDU	SOF, ROB, BFR, EMB, BSA
ER16	Pain detection†	Automatic assessment of pain intensity (rather than asking for subjective feelings) and its long-term monitoring through facial expression analysis. These clinic studies have attracted the interest of pharmaceutical companies (e.g. pain killer vendors), medical insurance companies, etc. It is also very useful to assess pain in patients that cannot communicate (e.g. due to Alzheimer disease).[165–167]	FD + AU (+FER)	CLI	PCK, BSA
ER17	Police interrogations	Analyse facial behavior and subtle reactions (facial micro-expressions), e.g. during police interrogations, court trials or border control interviews, in order to detect potential deception or affective states.[168–173]	FD + AU (+FER)	LE, MIG, JUS	DSC, HER, XIN, WSE
ER18	Emotion estimation in groups or crowds	Estimate the overall emotional state (e.g. global valence and arousal) of a medium-to-large group of people. The system might detect a panicked, calm or excited crowd, or to measure the degree of enjoyment of the crowd during a given event (e.g. a concert, footbal match, etc.).[174–178]	FD + FER	LE, VSU, ENT	-

Supplementary Table 4. List of 18 facial analysis applications under the category of Emotion Recognition (ER). For applications marked with †, the AI system could potentially be a safety component or part of a machine (e.g. car) or medical device.

#	Application	Description	Tasks	Areas	Companies
OT1	Pandemic control	Count and track people for pandemic control, to check the fulfilment of social distancing, occupancy rates, lockdown and other restrictions (without identifying persons).[179–182]	FD + FT	LE, VSU, CLI	HIK, HER, AVI, MAR, IPS, BFC, SAI
OT2	Occupancy control	Count and track the number of persons (faces) in a given indoor space (e.g. an airport gate, building entrance, museum room, restaurant, hospital, shop) to check its percent of occupancy. Can also be used for the control of queues (e.g. estimate waiting time and open new counters accordingly).[183–185]	FD + FT	MCI, VSU, MKT, TOU, ENT, CLI	HER, FFC, HIK, VIV, MIL, IPS, QUA, SIG, DBA, HBI, COG, VIX, ROC, GOR, AGV, VOC, RNE, FEL, PAC
OT3	People counting in crowds	Count (or estimate) the number of persons in dense crowds, e.g. during a public demonstration or event.[186–188]	FD (+FT)	LE, VSU	HIK, CAN, IPS, DBA, GOR, RAI
OT4	Photo-sketch matching	Identify a real person from a sketch (i.e. a drawing). The matching can be performed in a direct way from the sketch image, or using a sketch-to-image translation technique as an intermediate processing step.[189–193]	(FAM+) FV	LE, JUS	-
OT5	Interactive facial attribute manipulation	Manipulate facial attributes in an interactive way, e.g. by selecting the attribute to change (hair style, thickness of the lips, beard, etc.) and by varying intensities (aging effect, make-up effect, etc.). It might be exploited for entertainment, as a support for aesthetics, to help LE bodies refine a subject's portrait based on witnesses' description (facial composite), etc.[194–199]	FAM	ENT, LE, CLI, JUS	VME, FAP, MEI, PIV, LOR, LUX
OT6	Facial augmented reality	Render a full range of 2D/3D effects in real-time over detected feature points on human face. Such effects include: overlaid virtual content (e.g. adding bunny ears, hats, glasses, sparkles around the face) or deformations (e.g. fish eye, cartoon effects).[200–202]	FD + FLE	ENT	SNP, BAN, IMC, APT, MEI, PIV, LUX, PAC, VTE
OT7	Image and video anonymisa-tion	Use of face detection to pixelate faces in videos. Deepfakes can alternatively be used to replace real faces by synthetic anonymous faces.[203–205]	FD (+FAM)	LE, VSU, ENT	CEL, BAI, DID, EYD, SIG
OT8	Visual lifelogging as memory aid†	Record one's daily life from a wearable camera (e.g. egocentric camera, bodycam or smart glasses) and log facial events. E.g. it might be used to recover Alzheimer or prosopagnosia patients' deserted memories, by showing them the summary of seen familiar faces throughout the day. It might also target social network users or bloggers, by allowing to automatically obtain the best daily facial snaps/cuts to upload.[206–209]	FD + FT (+FI)	SOC, CLI, ENT	MIC, NAR, GOO, MAN
OT9	Video sum-marisation	Summarize automatically long video contents by considering as key cuts those where persons (i.e. faces or most expressive faces) appear. As a result, hours of video footage might be reduced to most relevant minutes or seconds, saving a great amount of time and storage space. It is particularly useful for quickly reviewing hours of surveillance videos. It might also be used to summarize news, movies, sports and internet videos.[210–213]	FD (+FT +FER)	LE, VSU, ENT	BFC, INN, HUA
OT10	Generation of deepfake videos	Applications that perform face-swapping in videos, where faces of a target individual are replaced by the faces of a donor individual. The process might include a "live" process to animate a person in a still photo from the donor's video to precisely match his or her facial movements and expressions. It might be used to breathing life into existing still photos of ancestors, historical figures, politicians and celebrities.[214–217]	FD (+FT) + FAM (+FER)	ENT, POL, MKT, CUL	DID, DIS, OXO
OT11	Facial motion capture for virtual character animation	Detect facial landmarks and/or AUs from a human face and use them to animate virtual characters. It might include lip reading technology to make characters speak.[218–222]	FD + FLE (+AU +ALR)	ENT	ADB, SNP, GEN, DRS, ILM, NEU, SEN, SPA, DIS, LUX, OXO
OT12	Evidence for parentage and kinship	Determine the probability of having a kin relationship between two individuals based on their facial similarity (e.g. mother-son, father-daughter, grandfather-grandson, siblings). Aging or gender manipulation might be used as an intermediate step for kin verification. The similarity of facial expression dynamics might be studied in the case of videos. This use case can also be applied in the context of fertility clinics, to select sperm or egg donor based on facial donor-recipient similarity.[223–227]	FD (+FAM +FER) + KV	LE, JUS, SOC, CLI	FEN
OT13	Automatic transcription or enhancement of speech†	Transcript or enhance speech contents in videos using ALR when the audio channel is not available or highly noisy, e.g. in video-surveillance videos or multimedia contents. It might also be used as a communication support system for the hearing impaired.[218, 228]	FD (+FLE) + ALR	LE, VSU, CLI, ENT	LIO, 121
OT14	Speech recognition for voice impaired†	Lip-reading system used as a communication aid for the voice impaired, after having a tracheotomy, stroke, trauma or other conditions.[229, 230]	FD (+FLE) + ALR	CLI	LIO, 121
OT15	Face-guided communica-tion and interaction†	Using facial movements (micro-expressions, head pose, etc.) as a means of interaction and/or communication. Examples include the use of the face as a joystick for gaming, for a disabled person to drive a motorized wheelchair (e.g. raising an eyebrow to move forward, sticking out tongue to stop, etc.) or to communicate daily needs (e.g. by choosing pictograms in a graphic interface).[231–233]	FD + FT (+AU +FER)	CLI, ENT	HOO, ITL, VTE

Supplementary Table 5. List of 15 facial analysis applications under the category Other (OT). For applications marked with †, the AI system could potentially be a safety component or part of a medical device.

Key companies on facial processing

List of companies

This section contains the list of 183 companies identified as key players in the field of facial processing, as of December 2021 and in alphabetical order. Each company has been assigned a 3-letters code for easier identification. Other information provided is the country of headquarters, main web page URL and company size (SME vs large).

Supplementary Table 6. List of 183 key AI companies worldwide performing research and development on facial processing and/or having related products in their portfolio.

Code	Company name	Country	URL	Type
121	121 Captions	UK	https://www.121captions.com	SME
3DI	3DiVi	Russia	https://face.3divi.com	SME
ADB	Adobe	USA	https://www.adobe.com	Large
AFF	Affectiva	USA	https://www.affectiva.com	SME
AGV	AllGoVision Technologies	India	https://www.allgovision.com	SME
AIM	A.I.Mergence	France	https://www.ai-mergence.com	SME
ALM	Almax	Italy	https://www.almax-italy.it	SME
AMA	Amazon	USA	https://aws.amazon.com/rekognition	Large
ANI	Animetrics	USA	http://animetrics.com	SME
ANK	Anki	USA	https://ankicozmorobot.com	SME
APL	Apple	USA	https://www.apple.com	Large
APT	Apptly	USA	http://www.appt.ly	SME
AVI	Anyvision	Israel	https://www.anyvision.co	SME
AWA	Aware	USA	https://www.aware.com	SME
AYO	Ayonix	Japan	https://ayonix.com	SME
BAI	Brighter AI	Germany	https://brighter.ai	SME
BAN	Banuba	Hong Kong	https://www.banuba.com	SME
BFC	BriefCam	USA	https://www.briefcam.com	SME
BFR	Blue Frog Robotics	France	http://www.bluefrogrobotics.com	SME
BID	BioID	Germany	https://www.bioid.com	SME
BSA	BlueSkeye AI	UK	https://www.blueskeye.com	SME
CAM	Camvi	USA	https://www.camvi.com	SME
CAN	Canon	Japan	https://global.canon	Large
CBL	CyberLink	Taiwan	https://www.cyberlink.com	Large
CCH	Clear Channel Scandinavia	Sweden	https://www.clearchannel.se/en/	Large
CCO	CaraCom	Finland	https://www.caracom.fi/en/	SME
CEL	Celantur	Austria	https://www.celantur.com	SME
CIV	Civic	USA	https://www.civic.com	SME
CLA	Clarifai	USA	https://www.clarifai.com	SME
CLE	Clearview AI	USA	https://www.clearview.ai/	SME
CLO	Cloudwalk	China	https://www.cloudwalk.com	SME
CLR	Clear	USA	https://www.clearme.com	Large
COG	Cognitec	Germany	https://www.cognitec.com	SME
CYB	CyberExtruder	USA	https://cyberextruder.com	SME
DAO	Daon	Ireland	https://www.daon.com	Large
DBA	Digital Barriers	UK	https://www.digitalbarriers.com	SME
DEG	DeepGlint	China	http://www.deepglint.com	SME
DER	Dermalog	Germany	https://www.dermalog.com	Large
DID	D-ID	Israel	https://www.d-id.com	SME
DIS	Disney	USA	https://studios.disneyresearch.com	Large
DON	Dongwo Technology	China	http://www.nombt.com/en/	Large
DRS	Dreamr Studios	Australia	https://www.dreamrstudios.com	SME
DSC	Discern Science	USA	https://www.discernscience.com	SME
ELH	Element Human	UK	https://www.elementhuman.com	SME
EMB	Embodied	USA	https://embodied.com	SME
EMO	Emosta	Japan	https://emosta.com	SME
EMT	Emotech	UK	https://www.emotech.ai	SME
EPR	Elephant Robotics	China	https://www.elephantrobotics.com	SME
ETI	Etiqmedia	Spain	http://etiqmedia.com	SME
ETT	Entropik Tech	India	https://entropiktech.com	SME
EVO	Evolve Security	UK	http://www.evolvesecurityproducts.com	SME
EXA	Examity	USA	https://www.examity.com	Large

Code	Company name	Country	URL	Type
EYA	Eyeris	USA	https://www.eyeris.ai	SME
EYD	Eyedea	Czechia	https://eyedea.ai	SME
FAP	FaceApp	Cyprus	https://www.faceapp.com	SME
FBK	Facebook	USA	https://www.facebook.com	Large
FEL	Felenasoft	Russia	https://felenasoft.com	SME
FEN	Fenomatch	Spain	https://fenomatch.com	SME
FFC	FootFallCam	UK	https://www.footfallcam.com	SME
FFR	FaceFirst	USA	https://www.facefirst.com	SME
FOR	Ford	USA	https://corporate.ford.com	Large
FPH	FacePhi	Spain	https://www.facephi.com	SME
FSA	Find Solution AI	Hong Kong	https://www.findsolutionai.com	SME
FUJ	Fujisoft	Japan	https://palro.jp	Large
FUL	Fulcrum Biometrics	USA	https://www.fulcrumbiometrics.com	SME
FUR	Furhat Robotics	Sweden	https://furhatrobotics.com	SME
GEN	Genies	USA	https://genies.com	SME
GMO	General Motors	USA	https://www.gm.com	Large
GOO	Google	USA	https://about.google	Large
GOR	Gorilla Technology	Taiwan	https://www.gorilla-technology.com	Large
HAN	Hanvon	China	https://www.hanvon.com	Large
HBI	HB Innovation	South Korea	http://hbinno.com	SME
HER	Herta Security	Spain	http://herta.ai	SME
HID	HID Global	USA	https://www.hidglobal.com	Large
HIK	Hikvision	China	https://www.hikvision.com	Large
HIS	Hisign	China	http://www.biometric.cn	Large
HLK	Honorlock	USA	https://honorlock.com	SME
HON	Honda	Japan	https://global.honda	Large
HOO	Hoobox Robotics	Brasil	https://hoobox.one	SME
HRV	HireVue	USA	https://www.hirevue.com	Large
HUA	Huawei	China	https://www.huawei.com	Large
HYU	Hyundai	South Korea	https://www.hyundai.com	Large
ICU	iCub Tech	Italy	https://www.iit.it/web/icub-tech	SME
ID3	ID3 Technologies	France	https://id3.eu	SME
IDE	IDEMIA	France	https://www.idemia.com	Large
IFL	Iflexion	USA	https://www.iflexion.com	SME
ILM	ILM	USA	https://www.ilm.com	Large
IMC	Imascono	Spain	https://imascono.com	SME
IMO	iMotions	Denmark	https://imotions.com	SME
INN	Innovatrics	Slovakia	https://www.innovatrics.com	SME
INT	IntelliVision	USA	https://www.intelli-vision.com	SME
IPS	Ipsotek	UK	https://www.ipsotek.com	SME
IRE	Irex	USA	https://irex.ai	SME
ISS	ISS	USA	https://issivs.com	Large
ITL	Intel	USA	https://www.intelrealsense.com	Large
JBO	Jibo	USA	https://jibo.com	SME
JIB	Jibble	Malaysia	https://www.jibble.io	SME
KAI	Kairos	USA	https://www.kairos.com	SME
KED	Kedacom	China	https://www.kedacom.com	Large
KIA	Kia	South Korea	https://www.kia.com	Large
KLE	KeyLemon	Switzerland	https://www.keylemon.com	SME
KOD	Kodak	USA	https://www.kodak.com	Large
LEX	Lexus	Japan	https://discoverlexus.com	Large
LIO	Liopa	UK	https://liopa.ai	SME
LLV	LLVision	China	https://www.llvision.com/en/	SME
LOR	L'Oréal	France	https://www.loreal.com	Large
LUX	Luxand	USA	https://www.luxand.com	SME
MAN	MannLab	Canada	https://mannlab.com	SME
MAR	Marlowe	UK	https://marlowefireandsecurity.com	SME
MCA	MorphCast	Italy	https://www.morphcast.com	SME
MEG	Megvii	China	https://en.megvii.com	Large
MEI	Meitu	China	https://www.meitu.com	Large
MIC	Microsoft	USA	https://www.microsoft.com	Large
MIL	Milestone	Denmark	https://www.milestonesys.com	Large
MOT	Motorola	USA	https://www.motorolasolutions.com	Large
NAR	Narrative	Sweden	http://getnarrative.com	SME
NEC	NEC	Japan	https://www.nec.com	Large
NEU	Neurotechnology	Lithuania	https://www.neurotechnology.com	SME
NIK	Nikon	Japan	https://www.nikon.com	Large
NOL	Noldus	Netherlands	https://www.noldus.com	SME

Code	Company name	Country	URL	Type
NTL	NtechLab	Russia	https://ntechlab.com	SME
NVI	Nviso	Switzerland	https://www.nviso.ai/en	SME
OLY	Olympus	Japan	https://www.olympus-global.com	Large
OMR	Omron	Japan	https://plus-sensing.omron.com	Large
OXA	Oxagile	USA	https://www.oxagile.com	Large
OXO	Oxolo	Germany	https://www.oxolo.com	SME
PAC	Packsee	China	https://www.panxsoft.com	SME
PAG	Pangian	USA	https://www.airportveriscan.com	SME
PAN	Panasonic	Japan	https://security.panasonic.com	Large
PAR	Paravision	USA	https://www.paravision.ai	SME
PCK	PainChek	Australia	www.painchek.com	SME
PCT	Proctorio	USA	https://proctorio.com	SME
PIV	PiVi Co	France	http://www.piviandco.com	SME
PRO	Pal Robotics	Spain	https://pal-robotics.com	SME
PRU	ProctorU	USA	https://www.proctoru.com	Large
PSO	Pixel Solutions	China	http://www.pixelall.com	SME
QUA	Quantasoft	Czechia	https://www.quantasoft.com	SME
RAI	Remark AI	USA	https://www.remarkholdings.com	SME
REN	Rendip	Singapore	https://www.rendip.com	SME
RES	Respondus	USA	https://web.respondus.com	SME
RET	Retorio	Germany	https://www.retorio.com	SME
REY	Real Eyes	UK	https://www.realeyesit.com	SME
RNE	Real Networks	USA	https://safr.com	Large
ROB	Robokind	USA	https://www.robokind.com	SME
ROC	Rank One Computing	USA	https://rankone.io	SME
SAI	Supervue AI	India	https://supervue.ai	SME
SAM	Samsung	South Korea	https://www.samsung.com	Large
SCV	ScanVis	Hong Kong	https://www.scanvis-ai.com	SME
SEC	SensumCo	UK	http://sensum.co	SME
SEE	Seeing Machines	Australia	https://www.seeingmachines.com	SME
SEN	Sense Time	China	https://www.sensetime.com	Large
SIG	Sightcorp	Netherlands	https://sightcorp.com	SME
SIT	SITA	Switzerland	https://www.sita.aero	Large
SNP	Snap Inc	USA	https://www.snap.com	Large
SOF	Softbank Robotics	Japan	https://www.softbankrobotics.com	Large
SON	Sony	Japan	https://www.sony.com	Large
SPA	Spatial	USA	https://spatial.io	SME
SSY	Sensory	USA	https://www.sensory.com	SME
SVI	Stereovision Imaging	USA	http://www.svisite.com	SME
TAW	Tawny	Germany	https://www.tawny.ai	SME
TEB	Techno Brain	United Arab Emirates	https://technobraingroup.com	Large
TEC	Tech5	Switzerland	https://tech5.ai	SME
TFA	TrueFace	USA	https://www.trueface.ai	SME
THA	Thales	France	https://www.thalesgroup.com	Large
TOS	Toshiba	Japan	https://www.toshiba.co.jp	Large
UNI	Uniphore	USA	https://www.uniphore.com	Large
VAL	Valeo	France	https://www.valeo.com	Large
VAT	Video Analysis Technologies	Russia	https://tevian.ru	SME
VBX	Vision-Box	Portugal	https://www.vision-box.com	Large
VIV	Vividi	Czechia	https://vividi.io	SME
VIX	VixVizion	Australia	https://www.vixvizion.com	SME
VLA	VisionLabs	Netherlands	https://visionlabs.ai	SME
VME	VisionMetric	UK	https://visionmetric.com	SME
VOC	Vocord	Russia	http://en.vocord.ru	SME
VTE	Visage Technologies	Sweden	https://visagetechnologies.com	SME
WOL	Wolfcom	USA	https://wolfcomusa.com	SME
WSE	We See	UK	https://www.wesee.com	SME
XFA	XForwardAI	China	https://www.xforwardai.com.cn	SME
XIA	Xiaomi	China	https://www.mi.com/global	Large
XIN	Xinktech	China	http://www.xinktech.com	SME
YIT	YITU Tech	China	https://www.yitutech.com/en	Large
ZKT	ZKTeco	China	https://www.zkteco.me	Large
ZOR	Zorarobotics	Belgium	https://zorarobotics.be	SME

Distribution of companies by size and country

Supplementary Figure 4 shows the distribution of companies by size (SME vs large) and country of headquarters. As it can be observed, companies are mostly located in the USA (59 companies), Europe (44 companies in EU27, 14 in the UK and 4 in Switzerland) and Asia (20 companies in China, 14 in Japan, 4 in South Korea, 3 in Hong Kong). India, which is positioned high on global charts tracking AI industry development and technology adoption[234], has however a comparatively small presence in facial processing (3 companies).

Overall, although large companies are present in this landscape, the majority of facial processing-related companies are SMEs (66%). Nevertheless, the ratio of large companies vs SMEs is different depending on the geographic location. USA has, in absolute terms, a stronger presence of large firms (23 out of 59), followed by China (12 out of 20) and Japan (12 out of 14). Europe's facial analysis industry is mostly dominated by SMEs (35 out of 44 companies are SMEs in EU27). With 14 identified companies, the UK makes up a large part of the the European trend, where all of them are SMEs.

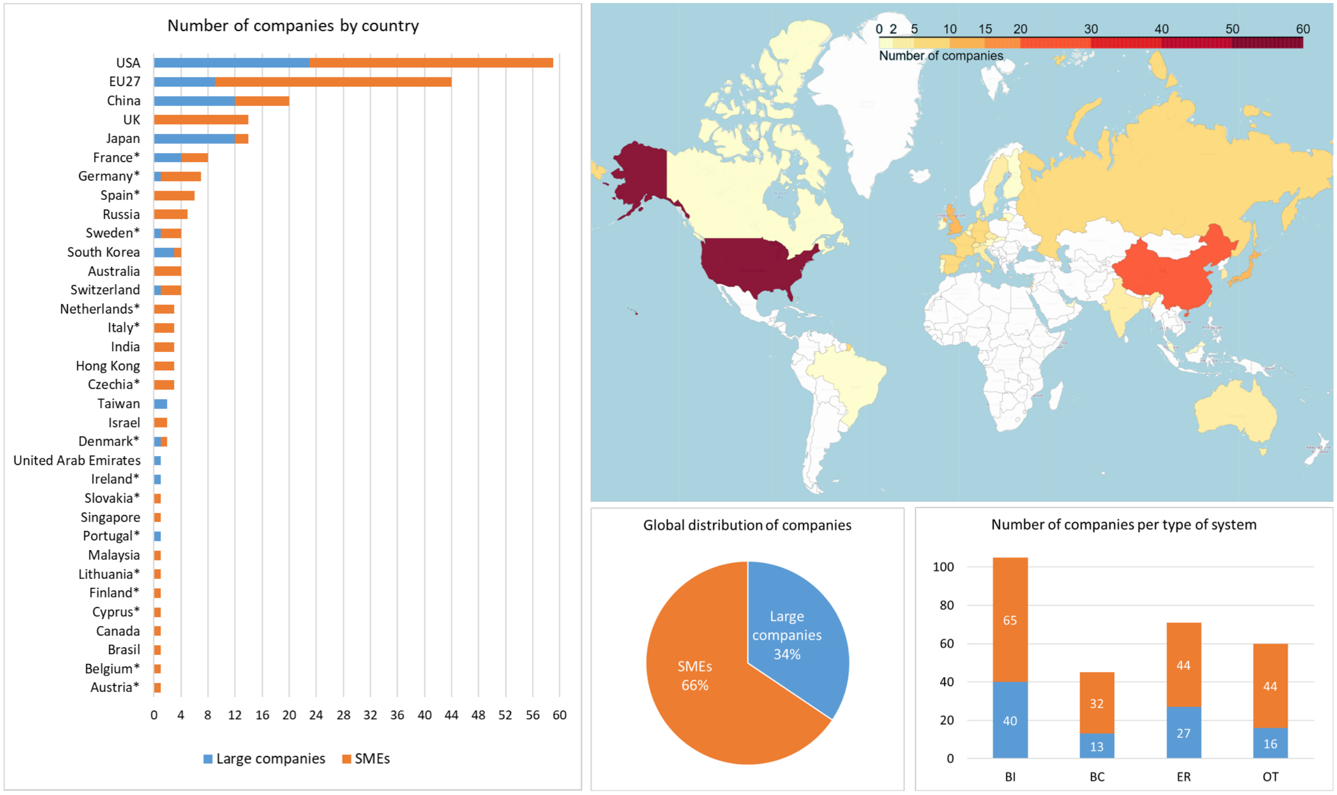

Supplementary Figure 4. Distribution of companies per country, size (SME vs large) and type of AI system commercialised (Biometric Identification - BI, Biometric Categorisation - BC, Emotion Recognition - ER or Other - OT). Countries marked with asterisk (*) belong to the EU27, which groups the 27 member countries of the European Union[235]. The world map was generated using the Folium v0.12.1 Python library (`https://python-visualization.github.io/folium/`).

References

1. Huang, G. B., Mattar, M., Berg, T. & Learned-Miller, E. Labeled faces in the wild: A database for studying face recognition in unconstrained environments. In *Workshop on faces in 'Real-Life' Images: detection, alignment, and recognition* (2008).
2. Wang, F. *et al.* The devil of face recognition is in the noise. In *Proceedings of the European Conference on Computer Vision (ECCV)*, 765–780 (2018).
3. Hupont, I. & Fernández, C. Demogpairs: Quantifying the impact of demographic imbalance in deep face recognition. In *14th IEEE International Conference on Automatic Face & Gesture Recognition (FG 2019)*, 1–7 (2019).
4. Guo, Y., Zhang, L., Hu, Y., He, X. & Gao, J. MS-Celeb-1M: A dataset and benchmark for large-scale face recognition. In *European Conference on Computer Vision*, 87–102 (Springer, 2016).
5. Zhu, Z. *et al.* Webface260m: A benchmark unveiling the power of million-scale deep face recognition. In *IEEE/CVF Conference on Computer Vision and Pattern Recognition*, 10492–10502 (2021).
6. Mavadati, S. M., Mahoor, M. H., Bartlett, K., Trinh, P. & Cohn, J. F. DISFA: A spontaneous facial action intensity database. *IEEE Transactions on Affect. Comput.* **4**, 151–160 (2013).
7. Zhang, X. *et al.* Bp4d-spontaneous: a high-resolution spontaneous 3d dynamic facial expression database. *Image Vis. Comput.* **32**, 692–706 (2014).
8. Mollahosseini, A., Hasani, B. & Mahoor, M. H. Affectnet: A database for facial expression, valence, and arousal computing in the wild. *IEEE Transactions on Affect. Comput.* **10**, 18–31 (2017).
9. Liu, Z., Luo, P., Wang, X. & Tang, X. Deep learning face attributes in the wild. In *International Conference on Computer Vision (ICCV)* (2015).
10. Liu, Z., Luo, P., Wang, X. & Tang, X. Deep learning face attributes in the wild. In *IEEE International Conference on Computer Vision*, 3730–3738 (2015).
11. Yang, S., Luo, P., Loy, C.-C. & Tang, X. Wider face: A face detection benchmark. In *IEEE Conference on Computer Vision and Pattern Recognition*, 5525–5533 (2016).
12. Yang, B., Yan, J., Lei, Z. & Li, S. Z. Fine-grained evaluation on face detection in the wild. In *11th IEEE International Conference on Automatic Face and Gesture Recognition (FG)* (2015).
13. Zhang, Y. *et al.* CelebA-Spoof: Large-Scale Face Anti-Spoofing Dataset with Rich Annotations. In *European Conference on Computer Vision (ECCV)* (2020).
14. Robinson, J. P., Shao, M., Wu, Y. & Fu, Y. Families in the Wild (FIW). *24th ACM Int. Conf. on Multimed.* (2016).
15. Afouras, T., Chung, J. S., Senior, A., Vinyals, O. & Zisserman, A. Deep audio-visual speech recognition. *IEEE transactions on pattern analysis machine intelligence* (2018).
16. Taigman, Y., Yang, M., Ranzato, M. & Wolf, L. Web-scale training for face identification. In *IEEE conference on Computer Vision and Pattern Recognition*, 2746–2754 (2015).
17. Schroff, F., Kalenichenko, D. & Philbin, J. Facenet: A unified embedding for face recognition and clustering. In *IEEE conference on Computer Vision and Pattern Recognition*, 815–823 (2015).
18. Ciaparrone, G. *et al.* Deep learning in video multi-object tracking: A survey. *Neurocomputing* **381**, 61–88 (2020).
19. Heusel, M., Ramsauer, H., Unterthiner, T., Nessler, B. & Hochreiter, S. Gans trained by a two time-scale update rule converge to a local nash equilibrium. *Adv. neural information processing systems* **30** (2017).
20. Dendorfer, P. *et al.* Motchallenge: A benchmark for single-camera multiple target tracking. *Int. J. Comput. Vis.* **129**, 845–881 (2021).
21. Lee, T.-W., Chiu, Y.-L., Wu, Y.-S. & Liu, H.-S. An intelligent image-based customer analysis service. In *16th IEEE Asia-Pacific Network Operations and Management Symposium* (2014).
22. Karim, N. T., Jain, S., Moonrinta, J., Dailey, M. N. & Ekpanyapong, M. Customer and target individual face analysis for retail analytics. In *IEEE International Workshop on Advanced Image Technology (IWAIT)*, 1–4 (2018).
23. Cao, Y.-T., Wang, J. & Tao, D. Symbiotic adversarial learning for attribute-based person search. In *European Conference on Computer Vision*, 230–247 (Springer, 2020).

24. Frikha, M., Fendri, E. & Hammami, M. People search based on attributes description provided by an eyewitness for video surveillance applications. *Multimed. Tools Appl.* **78**, 2045–2072 (2019).

25. Kumar, N., Berg, A., Belhumeur, P. N. & Nayar, S. Describable visual attributes for face verification and image search. *IEEE Transactions on Pattern Analysis Mach. Intell.* **33**, 1962–1977 (2011).

26. Galiyawala, H. J., Raval, M. S. & Laddha, A. Person retrieval in surveillance videos using deep soft biometrics. In *Deep Biometrics*, 191–214 (Springer, 2020).

27. Loey, M., Manogaran, G., Taha, M. H. N. & Khalifa, N. E. M. A hybrid deep transfer learning model with machine learning methods for face mask detection in the era of the covid-19 pandemic. *Measurement* **167**, 108288 (2021).

28. Nieto-Rodríguez, A., Mucientes, M. & Brea, V. M. System for medical mask detection in the operating room through facial attributes. In *Iberian Conference on Pattern Recognition and Image Analysis*, 138–145 (Springer, 2015).

29. Liu, S. & Agaian, S. S. Covid-19 face mask detection in a crowd using multi-model based on yolov3 and hand-crafted features. In *Multimodal Image Exploitation and Learning 2021*, vol. 11734, 117340M (International Society for Optics and Photonics, 2021).

30. Wang, Y. & Kosinski, M. Deep neural networks are more accurate than humans at detecting sexual orientation from facial images. *J. personality social psychology* **114**, 246 (2018).

31. Kosinski, M. Facial recognition technology can expose political orientation from naturalistic facial images. *Sci. reports* **11**, 1–7 (2021).

32. Liu, X. *et al.* Understanding beauty via deep facial features. In *IEEE/CVF Conference on Computer Vision and Pattern Recognition Workshops* (2019).

33. Wu, X. & Zhang, X. Automated inference on criminality using face images. *arXiv preprint arXiv:1611.04135* 4038–4052 (2016).

34. Wei, W. *et al.* Assessing facial symmetry and attractiveness using augmented reality. *Pattern Analysis Appl.* 1–17 (2021).

35. Gautam, R. & Mahara, P. Perceptive advertising using standardised facial features. In *IEEE International Conference on Digitization (ICD)*, 1–7 (2019).

36. Le, D.-N., Van Chung, L., Nguyen, G. N. *et al.* Performance evaluation of video-based face recognition approaches for online video contextual advertisement user-oriented system. In *Information Systems Design and Intelligent Applications*, 287–295 (Springer, 2016).

37. Yao, X., Chen, Y., Liao, R. & Cai, S. Face based advertisement recommendation with deep learning: A case study. In *International Conference on Smart Computing and Communication*, 96–102 (Springer, 2017).

38. Pasquale, L., Zippo, P., Curley, C., O'Neill, B. & Mongiello, M. Digital age of consent and age verification: Can they protect children? *IEEE Softw.* (2020).

39. Van Hoof, J. J. The effectiveness of id readers and remote age verification in enhancing compliance with the legal age limit for alcohol. *The Eur. J. Public Heal.* **27**, 357–359 (2017).

40. van Hoof, J. J. & van Velthoven, B. C. Remote age verification to prevent underage alcohol sales. first results from dutch liquor stores and the economic viability of national adoption. *Int. journal drug policy* **26**, 364–370 (2015).

41. Gurovich, Y. *et al.* Identifying facial phenotypes of genetic disorders using deep learning. *Nat. medicine* **25**, 60–64 (2019).

42. Yang, Y. A preliminary evaluation of still face images by deep learning: A potential screening test for childhood developmental disabilities. *Med. Hypotheses* **144**, 109978 (2020).

43. Kruszka, P. *et al.* Down syndrome in diverse populations. *Am. J. Med. Genet. Part A* **173**, 42–53 (2017).

44. Venkataraman, D. & Parameswaran, N. S. Extraction of facial features for depression detection among students. *Int. J. Pure Appl. Math* **118**, 455–463 (2018).

45. Januzaj, Y., Luma, A., Januzaj, Y. & Ramaj, V. Real time access control based on face recognition. In *International Conference on Network security & Computer Science (ICNSCS-15)*, 7–12 (2015).

46. Nag, A., Nikhilendra, J. & Kalmath, M. Iot based door access control using face recognition. In *IEEE 3rd International Conference for Convergence in Technology (I2CT)*, 1–3 (2018).

47. Lee, H., Park, S.-H., Yoo, J.-H., Jung, S.-H. & Huh, J.-H. Face recognition at a distance for a stand-alone access control system. *Sensors* **20**, 785 (2020).

48. Wang, Z. *et al.* Masked face recognition dataset and application. *arXiv preprint arXiv:2003.09093* (2020).

49. Ngan, M., Grother, P. & Hanaoka, K. Ongoing face recognition vendor test (frvt) part 6a: Face recognition accuracy with masks using pre- covid-19 algorithms (2020).

50. Li, C., Ge, S., Zhang, D. & Li, J. Look through masks: towards masked face recognition with de-occlusion distillation. In *28th ACM International Conference on Multimedia*, 3016–3024 (2020).

51. Carlos-Roca, L. R., Torres, I. H. & Tena, C. F. Facial recognition application for border control. In *IEEE International Joint Conference on Neural Networks (IJCNN)*, 1–7 (2018).

52. del Rio, J. S., Moctezuma, D., Conde, C., de Diego, I. M. & Cabello, E. Automated border control e-gates and facial recognition systems. *computers & security* **62**, 49–72 (2016).

53. Galdi, C. *et al.* Protect: Pervasive and user focused biometrics border project–a case study. *IET Biom.* **9**, 297–308 (2020).

54. Nasution, M. I. P., Nurbaiti, N., Nurlaila, N., Rahma, T. I. F. & Kamilah, K. Face recognition login authentication for digital payment solution at covid-19 pandemic. In *3rd IEEE International Conference on Computer and Informatics Engineering (IC2IE)*, 48–51 (2020).

55. Oliveira, J. S., Souza, G. B., Rocha, A. R., Deus, F. E. & Marana, A. N. Cross-domain deep face matching for real banking security systems. In *Seventh IEEE International Conference on eDemocracy & eGovernment (ICEDEG)*, 21–28 (2020).

56. Gusain, R., Jain, H. & Pratap, S. Enhancing bank security system using face recognition, iris scanner and palm vein technology. In *IEEE 3rd International Conference On Internet of Things: Smart Innovation and Usages (IoT-SIU)*, 1–5 (2018).

57. Mann, S. *et al.* Sensing of the self, society, and the environment. In *IEEE Sensors*, 1–4 (2020).

58. Welinder, Y. Facing real-time identification in mobile apps & wearable computers. *Santa Clara High Tech. LJ* **30**, 89 (2013).

59. Wang, Z., Cheng, Z., Huang, H., Zhou, X. & Liu, Y. Design and implementation of vehicle unlocking system based on face recognition. In *IEEE 34rd Youth academic annual conference of chinese association of automation (YAC)*, 121–126 (2019).

60. Olade, I., Liang, H.-n. & Fleming, C. A review of multimodal facial biometric authentication methods in mobile devices and their application in head mounted displays. In *IEEE SmartWorld, Ubiquitous Intelligence & Computing, Advanced & Trusted Computing, Scalable Computing & Communications, Cloud & Big Data Computing, Internet of People and Smart City Innovation*, 1997–2004 (2018).

61. Ahmed, S. U. *et al.* Security lock with effective verification traits. In *IEEE International Conference on Computational Intelligence and Knowledge Economy (ICCIKE)*, 164–169 (2019).

62. Prabhu, S. G. *et al.* Smart online voting system. In *7th IEEE International Conference on Advanced Computing and Communication Systems (ICACCS)*, vol. 1, 632–634 (2021).

63. Srikrishnaswetha, K., Kumar, S. & Mahmood, M. R. A study on smart electronics voting machine using face recognition and aadhar verification with IoT. In *Innovations in electronics and communication engineering*, 87–95 (Springer, 2019).

64. Purandare, H. V., Saini, A. R., Pereira, F. D., Mathew, B. & Patil, P. S. Application for online voting system using android device. In *IEEE International Conference on Smart City and Emerging Technology (ICSCET)*, 1–5 (2018).

65. Shovon, A. R., Roy, S., Sharma, T. & Whaiduzzaman, M. A restful e-governance application framework for people identity verification in cloud. In *International Conference on Cloud Computing*, 281–294 (Springer, 2018).

66. Barquero, G., Hupont, I. & Fernandez, C. Rank-based verification for long-term face tracking in crowded scenes. *IEEE Transactions on Biom. Behav. Identity Sci.* (2021).

67. Dhamecha, T. I., Shah, M., Verma, P., Vatsa, M. & Singh, R. Crowdfacedb: Database and benchmarking for face verification in crowd. *Pattern Recognit. Lett.* **107**, 17–24 (2018).

68. Klare, B. F. *et al.* Pushing the frontiers of unconstrained face detection and recognition: Iarpa Janus Benchmark A. In *IEEE Conference on Computer Vision and Pattern Recognition*, 1931–1939 (2015).

69. Ma, B., Su, Y. & Jurie, F. Bicov: a novel image representation for person re-identification and face verification. In *British Machive Vision Conference*, 11–pages (2012).

70. Bedagkar-Gala, A. & Shah, S. K. A survey of approaches and trends in person re-identification. *Image vision computing* **32**, 270–286 (2014).

71. Wu, D. *et al.* Deep learning-based methods for person re-identification: A comprehensive review. *Neurocomputing* **337**, 354–371 (2019).

72. Koide, K., Menegatti, E., Carraro, M., Munaro, M. & Miura, J. People tracking and re-identification by face recognition for rgb-d camera networks. In *IEEE European Conference on Mobile Robots (ECMR)*, 1–7 (2017).

73. Gallego, J. & Slater, M. Multi-person re-identification based on face, pose and texture analysis in unconstrained videos. In *IEEE 21st International Conference on Computational Problems of Electrical Engineering (CPEE)*, 1–4 (2020).

74. Jacquet, M. & Champod, C. Automated face recognition in forensic science: Review and perspectives. *Forensic Sci. Int.* **307**, 110124 (2020).

75. Salici, A. & Ciampini, C. Automatic face recognition and identification tools in the forensic science domain. In *International Tyrrhenian Workshop on Digital Communication*, 8–17 (Springer, 2017).

76. Mühling, M. *et al.* Deep learning for content-based video retrieval in film and television production. *Multimed. Tools Appl.* **76**, 22169–22194 (2017).

77. Tan, W. & Liu, J. Application of face recognition in tracing covid-19 fever patients and close contacts. In *IEEE 19th IEEE International Conference on Machine Learning and Applications (ICMLA)*, 1112–1116 (2020).

78. Berman, G., Carter, K., Herranz, M. & Sekara, V. Digital contact tracing and surveillance during covid-19. *Gen. child-specific ethical issues. UNICEF Off. Res.* (2020).

79. Kalra, I. *et al.* Dronesurf: Benchmark dataset for drone-based face recognition. In *14th IEEE International Conference on Automatic Face & Gesture Recognition (FG 2019)*, 1–7 (2019).

80. Hsu, H.-J. & Chen, K.-T. Face recognition on drones: Issues and limitations. In *First workshop on micro aerial vehicle networks, systems, and applications for civilian use*, 39–44 (2015).

81. Shanthi, K., Sivalakshmi, P. *et al.* Smart drone with real time face recognition. *Mater. Today: Proc.* (2021).

82. Sarath, R. N., Varghese, J. T. & Pandya, F. Unmanned aerial vehicle for human tracking using face recognition system. In *IEEE Advances in Science and Engineering Technology International Conferences (ASET)*, 1–5 (2019).

83. KH, A. B. *et al.* Smart cctv surveillance system for intrusion detection with live streaming. In *3rd IEEE International Conference on Recent Trends in Electronics, Information & Communication Technology (RTEICT)*, 1030–1035 (2018).

84. Sutabri, T., Pamungkur, A. K. & Saragih, R. E. Automatic attendance system for university student using face recognition based on deep learning. *Int. J. Mach. Learn. Comput.* **9**, 668–674 (2019).

85. Bhattacharya, S., Nainala, G. S., Das, P. & Routray, A. Smart attendance monitoring system (sams): a face recognition based attendance system for classroom environment. In *IEEE 18th International Conference on Advanced Learning Technologies (ICALT)*, 358–360 (2018).

86. Kaliappan, J., Shreyansh, J., Singamsetti, M. S. *et al.* Surveillance camera using face recognition for automatic attendance feeder and energy conservation in classroom. In *IEEE International Conference on Vision Towards Emerging Trends in Communication and Networking (ViTECoN)*, 1–5 (2019).

87. Tang, L. *et al.* Faces are protected as privacy: An automatic tagging framework against unpermitted photo sharing in social media. *IEEE Access* **7**, 75556–75567 (2019).

88. Wei, Z. *et al.* Autoprivacy: Automatic privacy protection and tagging suggestion for mobile social photo. *Comput. & Secur.* **76**, 341–353 (2018).

89. Balakrishnan, S., Chaudhuri, S. & Narasayya, V. Autotag'n search my photos: leveraging the social graph for photo tagging. In *24th International Conference on World Wide Web*, 163–166 (2015).

90. Tang, L. *et al.* My face is mine: Fighting unpermitted tagging on personal/group photos in social media. In *International Conference on Web Information Systems Engineering*, 528–539 (Springer, 2017).

91. Kianpisheh, M., Li, F. M. & Truong, K. N. Face recognition assistant for people with visual impairments. *ACM Conf. on Interactive, Mobile, Wearable Ubiquitous Technol.* **3**, 1–24 (2019).

92. Zhao, Y., Wu, S., Reynolds, L. & Azenkot, S. A face recognition application for people with visual impairments: Understanding use beyond the lab. In *CHI Conference on Human Factors in Computing Systems*, 1–14 (2018).

93. Lim, J., Yoo, Y., Cho, H. & Choi, S. Touchphoto: Enabling independent picture taking and understanding for visually-impaired users. In *International Conference on Multimodal Interaction*, 124–134 (2019).

94. Sandhya, B., Sahana, S. & Sneha, S. Third eye for blind. *Perspectives Commun. Embed. Signal-processing-PiCES* **4**, 280–283 (2021).

95. Paul, I. J. L. *et al.* Smart eye for visually impaired-an aid to help the blind people. In *2019 International Conference on Computational Intelligence in Data Science (ICCIDS)*, 1–5 (2019).

96. Agrawal, P. L. & Patil, D. Wearable face recognition system to aid visually impaired people. *Int. J. Sci. Res. Comput. Sci. Eng. Inf. Technol.* (2017).

97. Lee, K., Sato, D., Asakawa, S., Kacorri, H. & Asakawa, C. Pedestrian detection with wearable cameras for the blind: A two-way perspective. In *CHI Conference on Human Factors in Computing Systems*, 1–12 (2020).

98. Jin, Y., Kim, J., Kim, B., Mallipeddi, R. & Lee, M. Smart cane: Face recognition system for blind. In *3rd International Conference on Human-Agent Interaction*, 145–148 (2015).

99. Durmaz, B. & Ayday, E. Entering watch dogs*: Evaluating privacy risks against large-scale facial search and data collection. In *IEEE INFOCOM 2021-IEEE Conference on Computer Communications Workshops (INFOCOM WKSHPS)*, 1–6 (2021).

100. Wetzinger, E., Atanasov, M. & Kampel, M. Person profiling using image and facial attributes analyses on unconstrained images retrieved from online sources. In *International Conference on Image Analysis and Recognition*, 55–62 (Springer, 2016).

101. de Andrade, N. N. G., Martin, A. & Monteleone, S. " all the better to see you with, my dear": Facial recognition and privacy in online social networks. *IEEE security & privacy* **11**, 21–28 (2013).

102. Karishma, A., KRISHNAN, K. A., Kiran, A., Dalin, E. & Shivaji, S. Smart office surveillance robot using face recognition. *Int. J. Mech. Prod. Eng. Res. Dev.* **8**, 725–734 (2018).

103. Dharmasena, T. & Abeygunawardhana, P. Design and implementation of an autonomous indoor surveillance robot based on raspberry pi. In *IEEE International Conference on Advancements in Computing (ICAC)*, 244–248 (2019).

104. Vinay, A. *et al.* Effective descriptors based face recognition technique for robotic surveillance systems. *Procedia Comput. Sci.* **133**, 968–975 (2018).

105. Garg, A., Dixit, D., Khanna, A. & Doss, S. Ui personalization using face recognition and machine learning. In *Proceedings of the International Conference on Innovative Computing & Communications (ICICC)* (2020).

106. Kang, J., Anderson, D. V. & Hayes, M. H. Face recognition for vehicle personalization with near infrared frame differencing. *IEEE Transactions on Consumer Electron.* **62**, 316–324 (2016).

107. Kak, S. F. & Mustafa, F. M. Smart home management system based on face recognition index in real-time. In *IEEE International Conference on Advanced Science and Engineering (ICOASE)*, 40–45 (2019).

108. Chen, J., Ou, Q., Chi, Z. & Fu, H. Smile detection in the wild with deep convolutional neural networks. *Mach. vision applications* **28**, 173–183 (2017).

109. An, L., Yang, S. & Bhanu, B. Efficient smile detection by extreme learning machine. *Neurocomputing* **149**, 354–363 (2015).

110. Zhang, K., Huang, Y., Wu, H. & Wang, L. Facial smile detection based on deep learning features. In *3rd IAPR Asian Conference on Pattern Recognition (ACPR)*, 534–538 (2015).

111. Ali, I. & Dua, M. Smile detection using data amalgamation. *Procedia Comput. Sci.* **167**, 979–986 (2020).

112. Wendin, K., Allesen-Holm, B. H. & Bredie, W. L. Do facial reactions add new dimensions to measuring sensory responses to basic tastes? *Food quality preference* **22**, 346–354 (2011).

113. Devís, B. A. & Pocoví, M. Experiential marketing: Searching for emotional connection with consumers in pos. In *Applying Neuroscience to Business Practice*, 63–81 (IGI Global, 2017).

114. Ciaccia, F. R. D. A. S. *et al.* Seat comfort evaluation using face recognition technology. In *Congress of the International Ergonomics Association*, 545–554 (Springer, 2018).

115. Spivak, I., Krepych, S., Faifura, V. & Spivak, S. Methods and tools of face recognition for the marketing decision making. In *IEEE International Scientific-Practical Conference Problems of Infocommunications, Science and Technology (PIC S&T)*, 212–216 (2019).

116. Tallapragada, V., Rao, N. A. & Kanapala, S. Emometric: An iot integrated big data analytic system for real time retail customer's emotion tracking and analysis. *Int. J. Comput. Intell. Res.* **13**, 673–695 (2017).

117. Okada, G., Masui, K. & Tsumura, N. Advertisement effectiveness estimation based on crowdsourced multimodal affective responses. In *IEEE Conference on Computer Vision and Pattern Recognition workshops*, 1263–1271 (2018).

118. McDUFF, D. & Berger, J. Why do some advertisements get shared more than others?: Quantifying facial expressions to gain new insights. *J. Advert. Res.* **60**, 370–380 (2020).

119. Lebreton, P. *et al.* Crowdwatcher: an open-source platform to catch the eye of the crowd. *Qual. User Exp.* **4**, 1–17 (2019).

120. Manullang, M. C. T., Ramdani, A. L. & Migotuwio, N. Design and architecture of a public satisfaction detection camera based on facial emotional analysis. In *IOP Conference Series: Earth and Environmental Science*, vol. 537, 012021 (IOP Publishing, 2020).

121. Altieri, A. *et al.* Affective guide for museum: A system to suggest museum paths based on visitors' emotions. In *International Conference on Human-Computer Interaction*, 521–532 (Springer, 2021).

122. Wei, H. *et al.* Relationship between environmental factors and facial expressions of visitors during the urban forest experience. *Urban For. & Urban Green.* **53**, 126699 (2020).

123. Baldassarri, S., Hupont, I., Abadía, D. & Cerezo, E. Affective-aware tutoring platform for interactive digital television. *Multimed. Tools Appl.* **74**, 3183–3206 (2015).

124. Han, L., Li, Y., Zhou, Z. & Song, P. Teaching effect analysis based on the facial expression recognition in classroom. *Mod. distance education research* **24**, 97–103 (2017).

125. Sawyer, R., Smith, A., Rowe, J., Azevedo, R. & Lester, J. Enhancing student models in game-based learning with facial expression recognition. In *Proceedings of the 25th conference on user modeling, adaptation and personalization*, 192–201 (2017).

126. Emerson, A., Cloude, E. B., Azevedo, R. & Lester, J. Multimodal learning analytics for game-based learning. *Br. J. Educ. Technol.* **51**, 1505–1526 (2020).

127. Hemamou, L., Felhi, G., Martin, J.-C. & Clavel, C. Slices of attention in asynchronous video job interviews. In *8th International Conference on Affective Computing and Intelligent Interaction (ACII)*, 1–7 (2019).

128. Su, Y.-S., Suen, H.-Y. & Hung, K.-E. Predicting behavioral competencies automatically from facial expressions in real-time video-recorded interviews. *J. Real-Time Image Process.* 1–11 (2021).

129. Suen, H.-Y., Hung, K.-E. & Lin, C.-L. Tensorflow-based automatic personality recognition used in asynchronous video interviews. *IEEE Access* **7**, 61018–61023 (2019).

130. Murali, P. *et al.* Affectivespotlight: Facilitating the communication of affective responses from audience members during online presentations. In *CHI 2021* (2021).

131. Navarathna, R., Carr, P., Lucey, P. & Matthews, I. Estimating audience engagement to predict movie ratings. *IEEE Transactions on Affect. Comput.* **10**, 48–59 (2017).

132. Sun, W., Li, Y., Tian, F., Fan, X. & Wang, H. How presenters perceive and react to audience flow prediction in-situ: An explorative study of live online lectures. *ACM Conf. on Human-Computer Interact.* **3**, 1–19 (2019).

133. Wu, H., Liu, Y., Liu, Y. & Liu, S. Fast facial smile detection using convolutional neural network in an intelligent working environment. *Infrared Phys. & Technol.* **104**, 103061 (2020).

134. Setiono, D., Saputra, D., Putra, K., Moniaga, J. V. & Chowanda, A. Enhancing player experience in game with affective computing. *Procedia Comput. Sci.* **179**, 781–788 (2021).

135. Akbar, M. T. *et al.* Enhancing game experience with facial expression recognition as dynamic balancing. *Procedia Comput. Sci.* **157**, 388–395 (2019).

136. Robinson, R., Wiley, K., Rezaeivahdati, A., Klarkowski, M. & Mandryk, R. L. "let's get physiological, physiological!" a systematic review of affective gaming. In *Annual Symposium on Computer-Human Interaction in Play*, 132–147 (2020).

137. Hupont, I., Cerezo, E., Baldassarri, S. & Del-Hoyo, R. Emotracking digital art. In *International Conference on Human-Computer Interaction*, 739–749 (Springer, 2014).

138. Duan, Y., Zhang, J. & Gu, X. A novel paradigm to design personalized derived images of art paintings using an intelligent emotional analysis model. *Front. Psychol.* **12** (2021).

139. Rajcic, N. & McCormack, J. Mirror ritual: An affective interface for emotional self-reflection. In *Proceedings of the 2020 CHI Conference on Human Factors in Computing Systems*, 1–13 (2020).

140. Visnu Dharsini, S., Balaji, B., Kirubha Hari, K. *et al.* Music recommendation system based on facial emotion recognition. *J. Comput. Theor. Nanosci.* **17**, 1662–1665 (2020).

141. Bielozorov, A., Bezbradica, M. & Helfert, M. The role of user emotions for content personalization in e-commerce: literature review. In *International Conference on Human-Computer Interaction*, 177–193 (Springer, 2019).

142. Babanne, V., Borgaonkar, M., Katta, M., Kudale, P. & Deshpande, V. Emotion based personalized recommendation system. *Int. Res. J. Eng. Technol.* **7** (2020).

143. Gilda, S., Zafar, H., Soni, C. & Waghurdekar, K. Smart music player integrating facial emotion recognition and music mood recommendation. In *IEEE International Conference on Wireless Communications, Signal Processing and Networking (WiSPNET)*, 154–158 (2017).

144. Kumar, A. & Patra, R. Driver drowsiness monitoring system using visual behaviour and machine learning. In *IEEE Symposium on Computer Applications & Industrial Electronics (ISCAIE)*, 339–344 (2018).

145. Vural, E. *et al.* Drowsy driver detection through facial movement analysis. In *International Workshop on Human-Computer Interaction*, 6–18 (Springer, 2007).

146. Ihme, K., Dömeland, C., Freese, M. & Jipp, M. Frustration in the face of the driver: a simulator study on facial muscle activity during frustrated driving. *Interact. Stud.* **19**, 487–498 (2018).

147. Govindarajan, V., Driggs-Campbell, K. & Bajcsy, R. Affective driver state monitoring for personalized, adaptive adas. In *2018 21st International Conference on Intelligent Transportation Systems (ITSC)*, 1017–1022 (2018).

148. Fridman, L. Human-centered autonomous vehicle systems: Principles of effective shared autonomy. *arXiv preprint arXiv:1810.01835* (2018).

149. Sini, J., Marceddu, A. C. & Violante, M. Automatic emotion recognition for the calibration of autonomous driving functions. *Electronics* **9**, 518 (2020).

150. Salam, H., Celiktutan, O., Hupont, I., Gunes, H. & Chetouani, M. Fully automatic analysis of engagement and its relationship to personality in human-robot interactions. *IEEE Access* **5**, 705–721 (2016).

151. Sönmez, E. B., Köse, H. & Barkana, D. E. Towards a new computational affective system for personal assistive robots. In *28th Signal Processing and Communications Applications Conference (SIU)*, 1–4 (2020).

152. Baltanas, S.-F., Ruiz-Sarmiento, J.-R. & Gonzalez-Jimenez, J. A face recognition system for assistive robots. In *3rd International Conference on Applications of Intelligent Systems*, 1–6 (2020).

153. Faraj, Z. *et al.* Facially expressive humanoid robotic face. *HardwareX* **9**, e00117 (2021).

154. Cerezo, E. *et al.* Real-time facial expression recognition for natural interaction. In *Iberian Conference on Pattern Recognition and Image Analysis*, 40–47 (Springer, 2007).

155. DiPaola, S. & Yalçin, Ö. N. A multi-layer artificial intelligence and sensing based affective conversational embodied agent. In *IEEE 8th International Conference on Affective Computing and Intelligent Interaction Workshops and Demos (ACIIW)*, 91–92 (2019).

156. Martinez-Martin, E. & del Pobil, A. P. Personal robot assistants for elderly care: An overview. *Pers. assistants: Emerg. computational technologies* 77–91 (2018).

157. Lee, S. B. & Yoo, S. H. Design of the companion robot interaction for supporting major tasks of the elderly. In *IEEE 14th International Conference on Ubiquitous Robots and Ambient Intelligence (URAI)*, 655–659 (2017).

158. Abdollahi, H., Mollahosseini, A., Lane, J. T. & Mahoor, M. H. A pilot study on using an intelligent life-like robot as a companion for elderly individuals with dementia and depression. In *IEEE-RAS 17th International Conference on Humanoid Robotics (Humanoids)*, 541–546 (2017).

159. Ozdemir, D., Cibulka, J., Stepankova, O. & Holmerova, I. Design and implementation framework of social assistive robotics for people with dementia - A scoping review. *Heal. Technol.* 1–12 (2021).

160. Pour, A. G., Taheri, A., Alemi, M. & Meghdari, A. Human–robot facial expression reciprocal interaction platform: case studies on children with autism. *Int. J. Soc. Robotics* **10**, 179–198 (2018).

161. Huijnen, C. A., Lexis, M. A. & de Witte, L. P. Matching robot kaspar to autism spectrum disorder (asd) therapy and educational goals. *Int. J. Soc. Robotics* **8**, 445–455 (2016).

162. Wood, L. J., Zaraki, A., Robins, B. & Dautenhahn, K. Developing kaspar: a humanoid robot for children with autism. *Int. J. Soc. Robotics* **13**, 491–508 (2021).

163. Alnajjar, F., Cappuccio, M., Mubin, O., Arshad, R. & Shahid, S. Humanoid robots and autistic children: a review on technological tools to assess social attention and engagement. *Int. J. Humanoid Robotics* (2020).

164. Landowska, A. & Robins, B. Robot eye perspective in perceiving facial expressions in interaction with children with autism. In *AINA Workshops*, 1287–1297 (2020).

165. Lucey, P. *et al.* Automatically detecting pain in video through facial action units. *IEEE Transactions on Syst. Man, Cybern. Part B (Cybernetics)* **41**, 664–674 (2010).

166. Rojo, R., Prados-Frutos, J. C. & López-Valverde, A. Pain assessment using the facial action coding system. a systematic review. *Medicina Clin. (English Ed.* **145**, 350–355 (2015).

167. Bargshady, G. *et al.* Enhanced deep learning algorithm development to detect pain intensity from facial expression images. *Expert. Syst. with Appl.* **149**, 113305 (2020).

168. Avola, D., Cinque, L., Foresti, G. L. & Pannone, D. Automatic deception detection in rgb videos using facial action units. In *13th International Conference on Distributed Smart Cameras*, 1–6 (2019).

169. Owayjan, M., Kashour, A., Al Haddad, N., Fadel, M. & Al Souki, G. The design and development of a lie detection system using facial micro-expressions. In *2nd international conference on advances in computational tools for engineering applications (ACTEA)*, 33–38 (2012).

170. Wu, Z., Singh, B., Davis, L. & Subrahmanian, V. Deception detection in videos. In *AAAI Conference on Artificial Intelligence*, vol. 32 (2018).

171. Sánchez-Monedero, J. & Dencik, L. The politics of deceptive borders:'biomarkers of deceit'and the case of iborderctrl. *Information, Commun. & Soc.* 1–18 (2020).

172. Preu, M. S. *Emotional detection in automated border control: a discourse analysis of the case iBorderCtrl, 2016-2019*. B.S. thesis, University of Twente (2021).

173. Watson, H. iborderctrl: Exploring the issue of algorithmic bias in global governance. *Centre for Glob. Const.* (2020).

174. Urizar, O. J., Barakova, E. I., Marcenaro, L., Regazzoni, C. S. & Rauterberg, M. Emotion estimation in crowds: A survey. In *8th International Conference of Pattern Recognition Systems (ICPRS 2017)* (2017).

175. Sánchez, F. L., Hupont, I., Tabik, S. & Herrera, F. Revisiting crowd behaviour analysis through deep learning: Taxonomy, anomaly detection, crowd emotions, datasets, opportunities and prospects. *Inf. Fusion* (2020).

176. Rabiee, H. *et al.* Emotion-based crowd representation for abnormality detection. *arXiv preprint arXiv:1607.07646* (2016).

177. Guo, X., Zhu, B., Polanía, L. F., Boncelet, C. & Barner, K. E. Group-level emotion recognition using hybrid deep models based on faces, scenes, skeletons and visual attentions. *20th ACM Int. Conf. on Multimodal Interact.* 635–639 (2018).

178. Dhall, A., Kaur, A., Goecke, R. & Gedeon, T. Emotiw 2018: Audio-video, student engagement and group-level affect prediction. In *20th ACM International Conference on Multimodal Interaction*, 653–656 (2018).

179. Kolhar, M., Al-Turjman, F., Alameen, A. & Abualhaj, M. M. A three layered decentralized iot biometric architecture for city lockdown during covid-19 outbreak. *IEEE Access* **8**, 163608–163617 (2020).

180. Yadav, S. Deep learning based safe social distancing and face mask detection in public areas for covid-19 safety guidelines adherence. *Int. J. for Res. Appl. Sci. Eng. Technol.* **8**, 1368–1375 (2020).

181. Nguyen, C. T. *et al.* Enabling and emerging technologies for social distancing: a comprehensive survey and open problems. *arXiv preprint arXiv:2005.02816* (2020).

182. Nguyen, C. T. *et al.* A comprehensive survey of enabling and emerging technologies for social distancing—part ii: Emerging technologies and open issues. *IEEE Access* **8**, 154209–154236 (2020).

183. Sun, K., Zhao, Q. & Zou, J. A review of building occupancy measurement systems. *Energy Build.* **216**, 109965 (2020).

184. Chen, T.-Y., Chen, C.-H., Wang, D.-J. & Kuo, Y.-L. A people counting system based on face-detection. In *Fourth International Conference on Genetic and Evolutionary Computing* (2010).

185. Yang, J. *et al.* Comparison of different occupancy counting methods for single system-single zone applications. *Energy Build.* **172**, 221–234 (2018).

186. Liu, Y., Shi, M., Zhao, Q. & Wang, X. Point in, box out: Beyond counting persons in crowds. In *IEEE/CVF Conference on Computer Vision and Pattern Recognition*, 6469–6478 (2019).

187. Wang, F., Sang, J., Wu, Z., Liu, Q. & Sang, N. Hybrid attention network based on progressive embedding scale-context for crowd counting. *arXiv preprint arXiv:2106.02324* (2021).

188. Wang, C., Zhang, H., Yang, L., Liu, S. & Cao, X. Deep people counting in extremely dense crowds. In *23rd ACM International Conference on Multimedia*, 1299–1302 (2015).

189. Tang, X. & Wang, X. Face photo recognition using sketch. In *IEEE International Conference on Image Processing*, vol. 1, I–I (2002).

190. Fang, Y., Deng, W., Du, J. & Hu, J. Identity-aware cyclegan for face photo-sketch synthesis and recognition. *Pattern Recognit.* **102**, 107249 (2020).

191. Hu, M. & Guo, J. Facial attribute-controlled sketch-to-image translation with generative adversarial networks. *EURASIP J. on Image Video Process.* **2020**, 1–13 (2020).

192. Mangla, F. U., Shahzad, M., Lali, M. I. & Bukhari, S. A. C. Sketch-based facial recognition: a weighted component-based approach (wcba). *Multimed. Tools Appl.* **79**, 27533–27553 (2020).

193. Yang, Y., Hossain, M. Z., Gedeon, T. & Rahman, S. S2fgan: Semantically aware interactive sketch-to-face translation. *arXiv preprint arXiv:2011.14785* (2020).

194. Lee, C.-H., Liu, Z., Wu, L. & Luo, P. Maskgan: Towards diverse and interactive facial image manipulation. In *IEEE/CVF Conference on Computer Vision and Pattern Recognition*, 5549–5558 (2020).

195. Tan, Z. *et al.* Michigan: Multi-input-conditioned hair image generation for portrait editing. *arXiv preprint arXiv:2010.16417* (2020).

196. Hou, X., Zhang, X., Shen, L., Lai, Z. & Wan, J. Guidedstyle: Attribute knowledge guided style manipulation for semantic face editing. *arXiv preprint arXiv:2012.11856* (2020).

197. Borges, A. d. F. S. & Morimoto, C. H. A virtual makeup augmented reality system. In *IEEE 21st Symposium on Virtual and Augmented Reality (SVR)*, 34–42 (2019).

198. He, Z., Zuo, W., Kan, M., Shan, S. & Chen, X. Attgan: Facial attribute editing by only changing what you want. *IEEE Transactions on Image Process.* **28**, 5464–5478 (2019).

199. Zaltron, N., Zurlo, L. & Risi, S. Cg-gan: An interactive evolutionary gan-based approach for facial composite generation. In *Proceedings of the AAAI Conference on Artificial Intelligence*, vol. 34, 2544–2551 (2020).

200. Kim, D.-J. Implementation of multi channel network platform based augmented reality facial emotion sticker using deep learning. *J. Digit. Contents Soc.* **19**, 1349–1355 (2018).

201. Wang, Z. & Yang, X. V-head: Face detection and alignment for facial augmented reality applications. In *International Conference on Multimedia Modeling*, 450–454 (Springer, 2017).

202. Hieda, N. & Cooperstock, J. R. Digital facial augmentation for interactive entertainment. In *IEEE 7th International Conference on Intelligent Technologies for Interactive Entertainment (INTETAIN)*, 9–16 (2015).

203. Maximov, M., Elezi, I. & Leal-Taixé, L. Ciagan: Conditional identity anonymization generative adversarial networks. In *IEEE/CVF Conference on Computer Vision and Pattern Recognition*, 5447–5456 (2020).

204. Hukkelås, H., Mester, R. & Lindseth, F. Deepprivacy: A generative adversarial network for face anonymization. In *International Symposium on Visual Computing*, 565–578 (Springer, 2019).

205. Ren, Z., Lee, Y. J. & Ryoo, M. S. Learning to anonymize faces for privacy preserving action detection. In *European Conference on Computer Vision (ECCV)*, 620–636 (2018).

206. Sultan, S. *et al.* A hybrid egocentric video summarization method to improve the healthcare for alzheimer patients. *J. Ambient Intell. Humaniz. Comput.* **10**, 4197–4206 (2019).

207. Doherty, A. R. & Smeaton, A. F. Combining face detection and novelty to identify important events in a visual lifelog. In *IEEE 8th International Conference on Computer and Information Technology Workshops*, 348–353 (2008).

208. Gurrin, C., Albatal, R., Joho, H. & Ishii, K. A privacy by design approach to lifelogging. In *Digital enlightenment yearbook 2014*, 49–73 (IOS Press, 2014).

209. Mann, S. *et al.* Face recognition and rehabilitation: A wearable assistive and training system for prosopagnosia. In *IEEE International Conference on Systems, Man, and Cybernetics (SMC)*, 731–737 (2020).

210. Jacob, H., Pádua, F. L., Lacerda, A. & Pereira, A. C. A video summarization approach based on the emulation of bottom-up mechanisms of visual attention. *J. Intell. Inf. Syst.* **49**, 193–211 (2017).

211. Haq, I. U. *et al.* Quicklook: Movie summarization using scene-based leading characters with psychological cues fusion. *Inf. Fusion* **76**, 24–35 (2021).

212. Khan, A. *et al.* Forensic video analysis: passive tracking system for automated person of interest (poi) localization. *IEEE Access* **6**, 43392–43403 (2018).

213. Yogameena, B. & Janani, R. Abnormal activity-based video synopsis by seam carving for atm surveillance applications. In *International Conference on Innovative Computing and Communications*, 453–462 (Springer, 2021).

214. Wiles, O., Koepke, A. & Zisserman, A. X2face: A network for controlling face generation using images, audio, and pose codes. In *European Conference on Computer Vision (ECCV)*, 670–686 (2018).

215. Mihailova, M. To dally with dalí: Deepfake (inter) faces in the art museum. *Convergence* 13548565211029401 (2021).

216. Naruniec, J., Helminger, L., Schroers, C. & Weber, R. M. High-resolution neural face swapping for visual effects. In *Computer Graphics Forum*, vol. 39, 173–184 (Wiley Online Library, 2020).

217. Nguyen, T. T., Nguyen, C. M., Nguyen, D. T., Nguyen, D. T. & Nahavandi, S. Deep learning for deepfakes creation and detection: A survey. *arXiv preprint arXiv:1909.11573* (2019).

218. Bhat, C., Ahmed, I., Saxena, V. & Kopparapu, S. K. Visual subtitles for internet videos. In *Fourth Workshop on Speech and Language Processing for Assistive Technologies*, 17–20 (2013).

219. Taylor, S. *et al.* A deep learning approach for generalized speech animation. *ACM Transactions on Graph. (TOG)* **36**, 1–11 (2017).

220. Intyanto, G., Yuniarti, D. & Pawening, A. Implement of the transformation for markerless facial motion capture in 3d animation. In *Journal of Physics: Conference Series*, vol. 1836, 012033 (IOP Publishing, 2021).

221. Gao, F. Unlabeled facial expression capture method in virtual reality system based on big data. *Int. J. Inf. Commun. Technol.* **18**, 261–274 (2021).

222. Barros, J. M. D., Golyanik, V., Varanasi, K. & Stricker, D. Face it!: A pipeline for real-time performance-driven facial animation. In *IEEE International Conference on Image Processing (ICIP)*, 2209–2213 (2019).

223. Georgopoulos, M., Panagakis, Y. & Pantic, M. Modeling of facial aging and kinship: A survey. *Image Vis. Comput.* **80**, 58–79 (2018).

224. Lu, J. *et al.* Kinship verification in the wild: The first kinship verification competition. In *IEEE International Joint Conference on Biometrics*, 1–6 (2014).

225. Dibeklioglu, H., Ali Salah, A. & Gevers, T. Like father, like son: Facial expression dynamics for kinship verification. In *IEEE International Conference on Computer Vision*, 1497–1504 (2013).

226. Wu, X., Boutellaa, E., Feng, X. & Hadid, A. Kinship verification from faces: Methods, databases and challenges. In *IEEE International Conference on Signal Processing, Communications and Computing (ICSPCC)*, 1–6 (2016).

227. Melendez, F., Gonzalez, S. & Villalba, L. A. La cara de la reproducción asistida. usando análisis facial en selección de donantes. the face of assisted reproduction. the use of facial analysis techniques on donor selection. *Revista Iberoamericana de Fertilidad* .

228. Adeel, A., Gogate, M. & Hussain, A. Towards next-generation lipreading driven hearing-aids: A preliminary prototype demo. In *International Workshop on Challenges in Hearing Assistive Technology (CHAT)*, vol. 19 (2017).

229. Kohlberg, G. D., Gal, Y. & Lalwani, A. K. Development of a low-cost, noninvasive, portable visual speech recognition program. *Annals otology, rhinology & laryngology* **125**, 752–757 (2016).

230. Gavras, S., Baxevanakis, S., Kikidis, D., Kyrodimos, E. & Exarchos, T. Towards a personalized multimodal system for natural voice reproduction. In *15th IEEE International Workshop on Semantic and Social Media Adaptation and Personalization (SMA*, 1–5 (2020).

231. Mahmud, S. *et al.* A multi-modal human machine interface for controlling a smart wheelchair. In *IEEE 7th Conference on Systems, Process and Control (ICSPC)*, 10–13 (2019).

232. Sharmila, A. Hybrid control approaches for hands-free high level human–computer interface-a review. *J. Med. Eng. & Technol.* **45**, 6–13 (2021).

233. Manresa-Yee, C., Varona, J., Perales, F. J. & Salinas, I. Design recommendations for camera-based head-controlled interfaces that replace the mouse for motion-impaired users. *Univers. access information society* **13**, 471–482 (2014).

234. Stanford University 2021 AI Index Report. https://hai.stanford.edu/research/ai-index-2021 (2021). [Online; accessed June 12, 2022].

235. EU27 country profiles. https://europa.eu/european-union/about-eu/countries_en. [Online; accessed June 12, 2022].
